# An intranasal ASO therapeutic targeting SARS-CoV-2

**DOI:** 10.1038/s41467-022-32216-0

**Published:** 2022-08-03

**Authors:** Chi Zhu, Justin Y. Lee, Jia Z. Woo, Lei Xu, Xammy Huu Wrynla, Livia H. Yamashiro, Fei Ji, Scott B. Biering, Erik Van Dis, Federico Gonzalez, Douglas Fox, Eddie Wehri, Arjun Rustagi, Benjamin A. Pinsky, Julia Schaletzky, Catherine A. Blish, Charles Chiu, Eva Harris, Ruslan I. Sadreyev, Sarah Stanley, Sakari Kauppinen, Silvi Rouskin, Anders M. Näär

**Affiliations:** 1 Department of Nutritional Sciences & Toxicology, University of California, Berkeley, CA USA; 2 Innovative Genomics Institute, University of California, Berkeley, CA USA; 3 Whitehead Institute for Biomedical Research, Cambridge, MA USA; 4 Department of Microbiology, Harvard Medical School, Boston, MA USA; 5 Division of Infectious Diseases and Vaccinology, School of Public Health, University of California, Berkeley, Berkeley, CA USA; 6 Department of Molecular and Cell Biology, Division of Immunology and Pathogenesis, University of California, Berkeley, CA USA; 7 Department of Molecular Biology, Massachusetts General Hospital, Boston, MA USA; 8 The Henry Wheeler Center for Emerging and Neglected Diseases, University of California, Berkeley, CA USA; 9 Department of Medicine, Division of Infectious Diseases and Geographic Medicine, Stanford University, School of Medicine, Stanford, CA USA; 10 Department of Pathology, Stanford University School of Medicine, Stanford, CA USA; 11 Department of Laboratory Medicine, University of California, San Francisco, CA USA; 12 Center for RNA Medicine, Aalborg University, Copenhagen, Denmark

**Keywords:** Drug development, Virology

## Abstract

The COVID-19 pandemic is exacting an increasing toll worldwide, with new SARS-CoV-2 variants emerging that exhibit higher infectivity rates and that may partially evade vaccine and antibody immunity. Rapid deployment of non-invasive therapeutic avenues capable of preventing infection by all SARS-CoV-2 variants could complement current vaccination efforts and help turn the tide on the COVID-19 pandemic. Here, we describe a novel therapeutic strategy targeting the SARS-CoV-2 RNA using locked nucleic acid antisense oligonucleotides (LNA ASOs). We identify an LNA ASO binding to the 5′ leader sequence of SARS-CoV-2 that disrupts a highly conserved stem-loop structure with nanomolar efficacy in preventing viral replication in human cells. Daily intranasal administration of this LNA ASO in the COVID-19 mouse model potently suppresses viral replication (>80-fold) in the lungs of infected mice. We find that the LNA ASO is efficacious in countering all SARS-CoV-2 “variants of concern” tested both in vitro and in vivo. Hence, inhaled LNA ASOs targeting SARS-CoV-2 represents a promising therapeutic approach to reduce or prevent transmission and decrease severity of COVID-19 in infected individuals. LNA ASOs are chemically stable and can be flexibly modified to target different viral RNA sequences and could be stockpiled for future coronavirus pandemics.

## Abstract (web summary)

Despite approved vaccines and anti-virals to prevent and treat SARS-CoV-2 infection, there is a need for further development of efficient antiviral therapeutic strategy. Here, Zhu et al. develop locked nucleic acid antisense oligonucleotides (LNA ASOs) targeting the 5’ leader sequence of SARS-CoV-2 RNA to interfere with replication of wildtype virus and variants of concern. Daily intranasal administration in K18-hACE2 humanized mice suppresses viral infection in lung.

## Introduction

The global coronavirus disease 19 (COVID-19) pandemic is caused by the highly pathogenic novel human SARS-coronavirus 2 (SARS-CoV-2)^1^. By contrast with previous outbreaks of related beta-coronaviruses (severe acute respiratory syndrome coronavirus 1 (SARS-CoV-1) and Middle East respiratory syndrome coronavirus (MERS-CoV)), SARS-CoV-2 infection causes lower mortality rate; however, the virus has a higher human-to-human transmission rate^2^, facilitating rapid spread across the world. As of July 2022, there are more than 546 million confirmed positive cases and in excess of 6.3 million reported deaths (WHO; www.who.int). Although vaccines are markedly slowing the increase in positive cases and deaths in some countries, many nations, especially in the developing world, do not have readily accessible vaccines^3^, and there is aversion among segments of the global population to vaccination^3^. Moreover, mutated variant strains of SARS-CoV-2 that evade immunity in response to previous infection or vaccination are continuously emerging, and are causing new local and global outbreaks^4,5^. Indeed, more than 80 variants have been identified to date, all with mutations in the Spike protein^6^, which SARS-CoV-2 utilizes for binding to the host cell-surface receptor angiotensin-converting enzyme 2 (ACE2) during host cell entry^7^. Several “variants of concern”, which harbor mutations in Spike that facilitate immune evasion and, in some cases, partial vaccine and monoclonal antibody therapeutics resistance, are spreading rapidly world-wide. These include B.1.1.7 (Alpha), B.1.351 (Beta), P.1 (Gamma), B.1.427/B.1.429 (Epsilon), B.1.617 (Kappa and Delta), and B.1.1.529 (Omicron) which are all more contagious and may in some cases lead to more severe disease^8^. The continuous evolution of the virus thus poses a daunting challenge to achieving “herd immunity”. Traditional drug screening and vaccine development is time intensive and may not be able to match the speed of emerging drug- or vaccine-resistant SARS-CoV-2 strains. There is clearly an urgent need for alternative approaches, including the rapid development of therapeutics that are active against all variants of concern.

To address these challenges, we developed a novel strategy to inhibit the replication of SARS-CoV-2 using locked nucleic acid-containing antisense oligonucleotides (LNA ASOs) targeting viral RNAs. LNA ASOs, which rely on Watson-Crick base-pairing to target specific complementary RNA sequences, can be quickly designed to target any viral or host RNA sequence, including non-coding structural elements that may be important for viral replication. Depending on the design, LNA ASOs may induce target RNA degradation through recruiting RNase H for cleavage (gapmers) or act through steric hindrance (mixmers) through high-affinity binding to complementary targets^9^. LNA ASOs are typically well tolerated, and a number of ASO therapeutics have been approved for clinical use^10^. Additionally, ASO manufacturing is well established and can be readily scaled-up. We have employed chemically modified gapmer and mixmer ASOs containing interspersed LNA and DNA nucleotides linked by phosphorothioate bonds. The introduced chemical modifications confer increased affinity, stability and improved pharmacokinetic/pharmacodynamic properties^11–13^.

SARS-CoV-2 is a compact (30 kilobases) positive-sense single-stranded RNA virus, with a 5’ untranslated region (UTR), the ORF1a/b RNA encoding non-structural viral proteins, and a 3’ segment encoding the structural proteins, such as the Spike protein that binds to the ACE2 receptor on host cells, and the nucleocapsid N protein involved in virion assembly, and a 3’ UTR^14^. The 5’ UTR, a non-coding segment consisting of multiple highly conserved stem-loops and more complex secondary structures, is functionally critical for viral translation and replication by affording protection from host cell antiviral defenses and through selective promotion of viral transcript translation over those of the host cell, at least in part through the recruitment of the viral non-structural protein 1 (Nsp1)^15^. The 5’ UTR begins with a short 5’ leader sequence (nucleotides 1-69), which is added via discontinuous transcription to the 5' end of all sub-genomic RNA transcripts encoding the viral structural proteins, and regulates their translation as well as translation of ORF1a/b from full-length genomic RNA^16^. The ORF1a/b also contains a structured and highly conserved frameshift stimulation element (FSE) near its center that controls a shift in the protein translation reading frame by one nucleotide of ORF1a/b genes 3’ to the FSE. The FSE and accurate frame shifting is crucial for the expression of ORF1b, which encodes five non-structural proteins including an RNA-dependent RNA polymerase (RdRP) essential for SARS-CoV-2 genome replication^17^.

In this work, we carry out screening of LNA ASOs targeting sequences and RNA secondary structures the SARS-CoV-2 genomic RNA to identify inhibitors of viral replication. Our studies uncover a potent LNA ASO targeting the critical stem-loop 1 (SL1) structure in the 5’ leader sequence of SARS-CoV-2, and it exhibits high anti-viral efficacy at nanomolar level against all major variants of concern in human cells. Further in vivo studies in mouse and hamster COVID-19 disease models reveal that the intranasal administration of this LNA ASO represents a potent and promising therapeutic strategy against SARS-CoV-2 viral infection and pathological manifestations, and it demonstrates both prophylactic and post-infection therapeutic effects in vivo. Importantly, our work indicates that inhaled LNA ASOs can be applied to treat or prevent virus-induced respiratory diseases, and will stimulate efforts to develop LNA ASO-based prophylactics and therapeutics for other viral respiratory diseases with pandemic potential.

## Results

### In vitro screening revealed strong anti-viral LNA ASO candidates targeting the 5’ leader of SARS-CoV-2

**Fig. 1:**
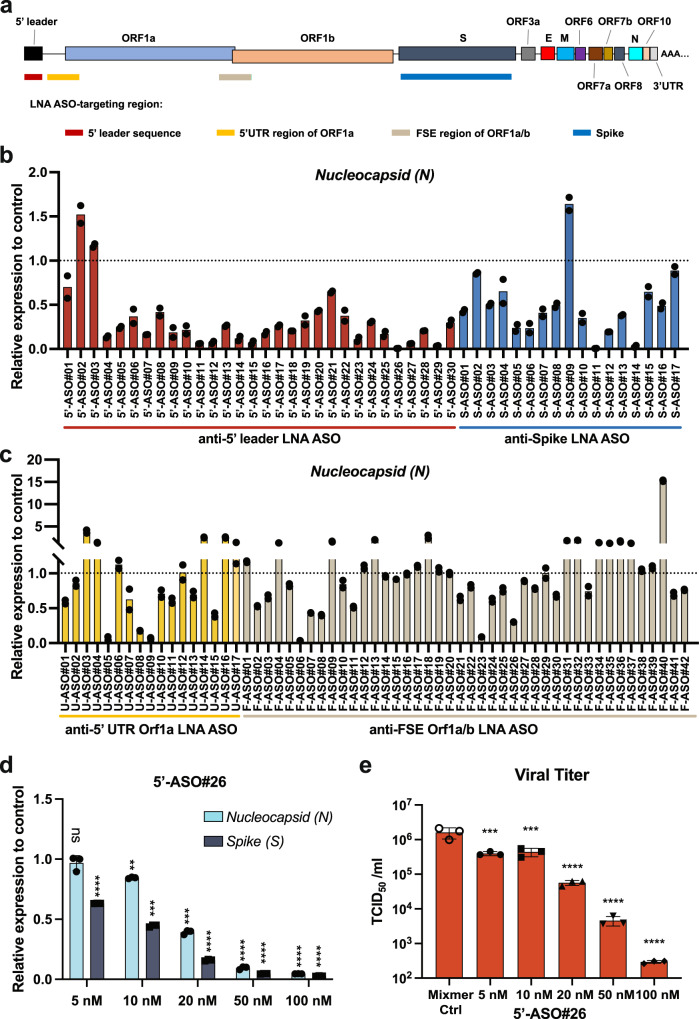
In vitro screening of LNA ASOs targeting SARS-CoV-2. **a** Schematic representation of the genome structure and the regions targeted by LNA ASOs. **b**, **c** The SARS-CoV-2 WA1 strain-infected Huh-7 cells treated with LNA ASO (100 nM) and cell culture medium were collected at 48 hpi. Viral RNA levels were analyzed by RT-qPCR for LNA ASO screening. Each LNA ASO was tested in duplicate and compared with in vitro Mixmer control LNA ASO or Gapmer control LNA ASO. **d** Dose-dependent effects of 5’-ASO#26 were evaluated in infected Huh-7 cells with increasing doses (*N* = 3) of the LNA ASO (as indicated) by RT-qPCR. The exact p-values stated in the following order: Nucleocapsid group and then Spike group ((Mixmer Ctrl vs. 5’_ASO#26), at 5 nM: *P* = 0.8279, **** *P* < 0.0001; at 10 nM: **P = 0.0012, *** *P* = 0.0002; at 20 nM: ***P = 0.0007, ****P < 0.0001; at 50 nM: *** *P* = 0.0002, **** *P* < 0.0001; at 100 nM: **** *P* < 0.0001, **** *P* < 0.0001. **e** The infectious virus was measured by TCID_50_ assay. The infected Huh-7 cells with different doses (*N* = 3) of LNA ASO treatment were collected at 48 hpi. The exact p-values stated here: at 5 nM, *** *P* = 0.0002; at 10 nM, *** *P* = 0.0003; at 20 nM, **** *P* < 0.0001; at 50 nM, **** *P* < 0.0001; at 100 nM, **** *P* < 0.0001. For **d** and **e**, one-way ANOVA with Dunnett’s test was used to determine significance (** *P* < 0.01, *** *P* < 0.001, **** *P* < 0.0001, *P* > 0.05, ns, not significant). Source data are provided as a Source Data file.

We designed multiple LNA ASOs targeting the 5’ leader sequence, downstream sequences in the 5’ untranslated region (UTR) of ORF1a/b, and the ORF1a/b FSE of SARS-CoV-2 (Fig. 1a and Supplementary Table 1). Additionally, given the importance of the Spike protein in host cell entry for SARS-CoV-2 infection, LNA ASOs targeting the Spike coding sequence were also tested (Fig. 1a and Supplementary Table 1). To evaluate the viral repressive effect of LNA ASOs, the initial screening was carried out in Huh-7 human hepatoma cells, which exhibit excellent transfection efficiency and are readily infected by SARS-CoV-2. Cells transfected with LNA ASOs were infected with SARS-CoV-2 and both cells and medium were collected at 48h post-infection (hpi) for RNA extraction and infectious viral particle determination, respectively. The viral titer was measured by detecting the expression level/copy number of *Nucleocapsid* (*N*) and *Spike* (*S*) using reverse transcription (RT)-quantitative PCR (qPCR). The screening results showed that the treatment with certain LNA ASOs lead to a dramatic decrease of *N* and *S* expression (Fig. 1b and 1c, Supplementary Fig. 1a and 1b).

Interestingly, we found that LNA ASOs targeting the 5’ leader region of SARS-CoV-2 were particularly effective in suppressing viral RNA levels in infected cells (Fig. 1b and Supplementary Fig. 1a). This is consistent with the fact that the 5’ leader sequence is present in all viral RNA transcripts and is required for viral replication. The most potent LNA ASO targeting the 5’ leader, 5’-ASO#26, was selected for further investigation of its viral repression capability. The repressive effect of 5’-ASO#26 was demonstrated in a dose-dependent manner by measuring the expression level of viral RNAs (Fig. 1d) and by directly determining the viral titer of infectious particles with the Fifty-percent Tissue Culture Infectious Dose (TCID_50_) assay in Vero E6 African green monkey kidney epithelial cells (Fig. 1e).

### LNA ASO 5’-ASO#26 interrupts the secondary structure of SARS-CoV-2 5’ leader sequence

**Fig. 2:**
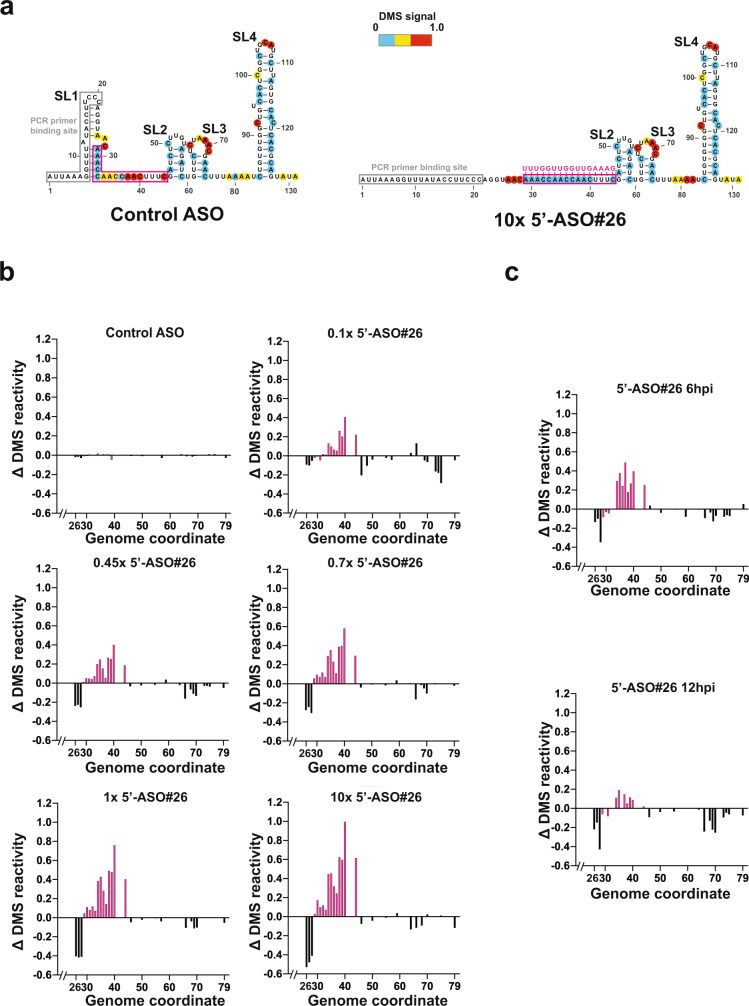
5’-ASO#26 disrupts stem loop 1 (SL1) of SARS-CoV-2. **a** Structural model of SL1-4 predicted from the DMS-MaPseq of in vitro-transcribed 5’leader RNA with the addition of control LNA ASO (left) and 5’-ASO#26 (right). Nucleotides are color-coded by normalized DMS signal. The 5’-ASO#26- sequence is shown in magenta and the binding site on the viral genome is highlighted with a pink frame; PCR primer binding site, where DMS information is unavailable, is highlighted with a grey frame. **b** Per-nucleotide-difference in DMS reactivity (Δ DMS reactivity) between in vitro-transcribed SARS-CoV-2 5’-leader RNA versus following the addition of control LNA ASO (top left), and versus titration with 5’ -ASO#26 at 0.1× (top right), 0.45× (middle left), 0.7× (middle right), 1× (bottom left) and 10× (bottom right) molar ratio of 5’-ASO#26. **c** Per-nucleotide-difference in DMS reactivity (Δ DMS reactivity) between Huh-7 cells transfected with control LNA ASO and those with 5’ -ASO#26, collected 6 (top) and 12 (bottom) hours after SARS-CoV-2 infection. For **b**, **c** only nucleotides from positions 26 to 79 in the SARS-CoV-2 genome are included. Each bar represents one nucleotide, and the 5’-ASO#26 target region is coloured in pink. Source data are provided as a Source Data file.

Although the 5’ UTR nucleotide sequences are somewhat divergent amongst the coronavirus family, the secondary structure of the 5’ UTR is highly conserved^18^, and it has been shown that two stem-loop structures, SL1 and SL2, are formed by the 5’ leader sequence^19^, which protect the viral RNA from Nsp1-mediated translational suppression^20^. Since the complementary sequence of 5’-ASO#26 aligns along the 3’ portion of SL1 (marked in pink frame) (Fig. 2a), we hypothesized that the viral repressive effect of 5’-ASO#26 may be in part due to its ability to disrupt the secondary structure of the 5’ leader sequence upon binding to the viral genomic or sub-genomic RNAs, interfering with the formation of the SL1 stem-loop structure. To test if 5’-ASO#26 can disrupt the SL1 structure, we carried out in vitro transcription of the SARS-CoV-2 5’ UTR sequence, added 5’-ASO#26 and treated samples with dimethyl sulfate (DMS). DMS is able to specifically and rapidly methylate unpaired adenines (A) and cytosines (C) within single-stranded sequences and not those that are complexed as RNA secondary structure or to the LNA ASO, allowing for the unpaired A or C nucleotides to be detected by DMS-MaPseq^21^. Our results strongly indicated that the secondary structure of SL1 was indeed interrupted by 5’-ASO#26 in a dose-dependent manner (Fig. 2b and Supplementary Fig. 2a). We also performed DMS-MaPseq with SARS-CoV-2-infected Huh-7 cells in the presence or absence of 5’-ASO#26, and monitored secondary structure changes of SL1 at 6 hpi and 12 hpi. Similar to the findings with the in vitro assay, the result from infected cells confirmed the disruption of the secondary structure of SL1 due to binding of 5’-ASO#26 (Fig. 2c and Supplementary Fig. 2b).

### Intranasal administration of 5’-ASO#26 did not induce significant immune stimulatory effect

**Fig. 3:**
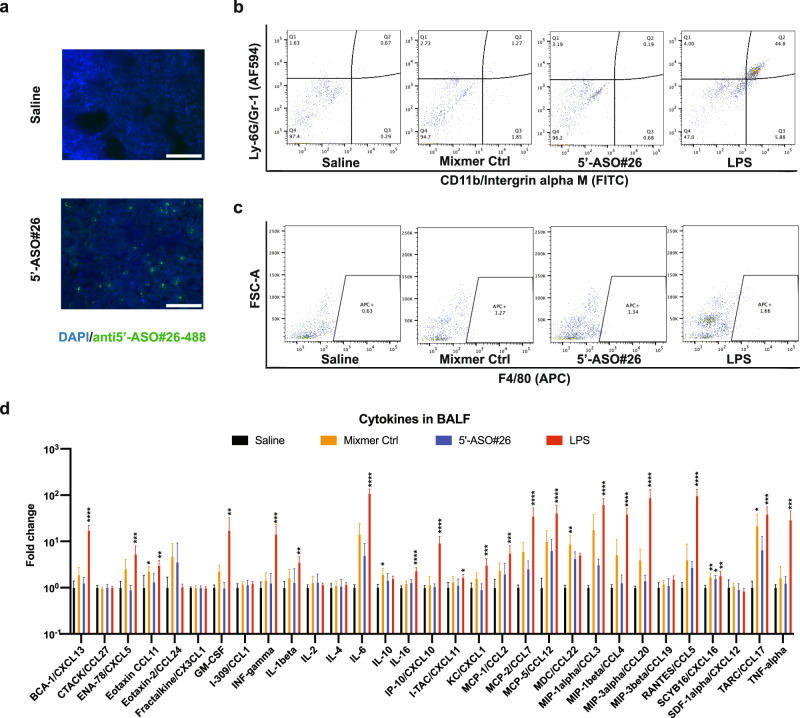
Evaluation of lung delivery efficacy and potential immune-stimulatory effects of LNA ASOs. **a** Representative 5’-ASO#26 FISH in mice lung. Scale bar = 100 μm. The FISH assay was repeated three times. **b**, **c** Representative flow cytometry result of alveolar neutrophils and macrophages in BALF collected from mice with three days-treatment of LNA ASOs or Saline; one day-treatment with LPS was used as a positive control. Neutrophils were identified as Ly-6G+ and CD11b+ and macrophages were identified as F4/80+. **d**) Multiplex cytokine assay in BALF collected from mice with three days-treatment of LNA ASOs or Saline (N = 5 for Saline, Mixmer Control and 5’-ASO#26 and N = 4 for LPS group, symbols represent mean ± SD). The exact p-values are: BCA-1/CXCL13(Saline vs. LPS), **** *P* < 0.0001; ENA-78/CXCL5(Saline vs. LPS), ***P < 0.001; CCL11(Saline vs. Mixmer Ctrl), * *P* = 0.0424; CCL11(Saline vs. LPS), **P = 0.0028; GM-CSF(Saline vs. LPS), **P = 0.0092; INF-gamma(Saline vs. LPS), ***P = 0.0005; IL-1beta(Saline vs. LPS), **P = 0.0036; IL-6(Saline vs. LPS), ****P < 0.0001; IL-10(Saline vs. Mixmer Ctrl), * *P* = 0.0286; IL-16(Saline vs. LPS), ****P < 0.0001; IP-10/CXCL10(Saline vs. LPS), ****P < 0.0001; I-TAC/CXCL11(Saline vs. LPS), * *P* = 0.0402; KC/CXCL1(Saline vs. LPS), *** *P* = 0.0004; MCP-1/CCL2(Saline vs. LPS), ***P = 0.0009; MCP-2/CCL7(Saline vs. LPS), ****P < 0.0001; MDC/CCL22(Saline vs. Mixmer Ctrl), **P = 0.0011; MIP-1alpha/CCL3(Saline vs. LPS), ****P < 0.0001, MIP-1beta/CCL4(Saline vs. LPS), **** *P* < 0.0001; MIP-1a/CCL20(Saline vs. LPS), ****P < 0.0001; RANTES/CCL5(Saline vs. LPS), ****P < 0.0001;SCYB16/CXCL16(Saline vs. Mixmer Ctrl), ** *P* = 0.0084; SCYB16/CXCL16(Saline vs. 5’-ASO#26), *P = 0.0341; SCYB16/CXCL16(Saline vs. LPS), **P = 0.0040; TARC/CCL17(Saline vs. Mixmer Ctrl), * *P* = 0.0448; TARC/CCL17(Saline vs. Mixmer Ctrl), *** *P* = 0.0009; TNF-alpha(Saline vs. LPS), ***P = 0.0001. For d), one-way ANOVA with Dunnett’s test was used to determine significance (* *P* < 0.05, ** *P* < 0.01, *** *P* < 0.001, **** *P* < 0.0001). Source data are provided as a Source Data file.

To investigate the effects of 5’-ASO#26 in vivo, we first evaluated the delivery of 5’-ASO#26 into mouse lung after intranasal administration. A previous study reported the efficient absorption of LNA ASO gapmers in lung after intratracheal administration, however predominantly induced macrophage recruitment and accumulation in liver and kidney^22^. In contrast, we found accumulation of 5’-ASO#26 in pulmonary epithelial cells after three days of daily intranasal treatment with 5’-ASO#26 using Fluorescence In Situ Hybridization (FISH) analysis (Fig. 3a). We speculate that the different absorption pattern of LNA ASOs may result from differences in the positioning of LNA-modified bases between gapmer and mixmer LNA ASOs, however this will need to be further investigated. Given the fact that lung is regarded as an immune organ and a previous study reported strong neutrophil recruitment and cytokine secretion in lung after intratracheal administration of LNA ASOs^23^, we evaluated the immune stimulatory effect of 5’-ASO#26 in the lung. In order to comprehensively study potential immune-stimulatory effects of 5’-ASO#26, we first carried out RNA-seq analysis of lung lobes that were collected from 5’-ASO#26- or control LNA ASO-treated C57BL/6J mice without SARS-CoV-2 infection. Only 6 and 106 genes were down- and up-regulated by 5’-ASO#26 treatment, respectively, whereas 2 and 35 genes were down- and up-regulated, respectively, by mixmer control LNA ASO in lung (Supplementary Table 2), with no overlap, suggesting no LNA ASO class effects. Moreover, only a few inflammation-related genes could be found in either up- or down-regulated genes in response to either 5’-ASO#26 or mixmer control treatments (Supplementary Table 2). Accordingly, we found no neutrophil or macrophage enrichment in either bronchoalveolar lavage fluid (BALF) (Fig. 3b, c and Supplementary Fig. 3a, Supplementary Fig. 4) or lung sections (Supplementary Fig. 3c). More importantly, consistent with the result of cell flow analysis, analysis of cytokine secretion in BALF of LNA ASO-treated mice indicated that there was no broad increase in cytokine secretion after LNA ASO treatments (Fig. 3d), especially IFN alpha remained at undetectable level after LNA ASO treatment (Supplementary Fig. 3b). Since the LNA ASOs used in previous studies were gapmers^23,24^ and 5’-ASO#26 is a mixmer, we believe that the lack of immune stimulatory effects of 5’-ASO#26 may be due to lower immunogenicity of LNA ASO mixmers, perhaps in addition to sequence-dependent effects^24^. Altogether, these findings support the non-immunogenicity and safety of the intranasally delivered mixmer LNA ASOs.

### 5’-ASO#26 exhibits in vivo anti-viral efficacy against SARS-CoV-2

**Fig. 4:**
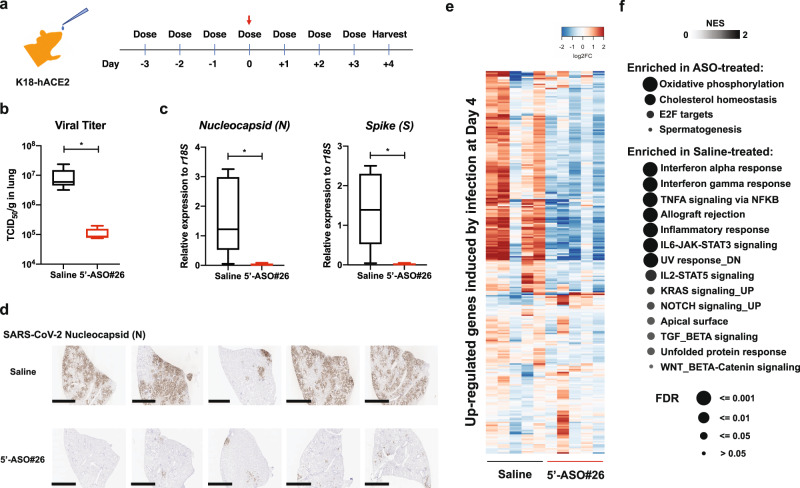
Once-daily treatment of 5′-ASO#26 represses the generation of infectious virus in mice. **a** Treatment course of once-daily 5’-ASO#26 administration. The red arrow indicates the inoculation of 1 × 10^4^ TCID50 SARS-CoV-2 intranasally in mice. Mice were treated once-daily with saline (vehicle) or 400 µg LNA ASO (dissolved in saline). Treatment on the day of infection was carried out at 6 hpi. **b** The viral burden in lungs of mice treated with Saline (*N* = 5) or LNA ASO (*N* = 5) was measured by TCID_50_ assay using lung homogenates. *P = 0.0427. Center line, median; box limits, upper and lower quartiles; plot limits, maximum and minimum in the boxplot. **c** The levels of viral RNAs encoding Nucleocapsid protein (N) (*P = 0.0246) and Spike (S) (*P = 0.0121) were measured in mouse lungs by RT-qPCR (*N* = 5). For **b** and **c**, center line, median; box limits, upper and lower quartiles; plot limits, maximum and minimum in the boxplot. Student *t*-test was used to determine significance (*P < 0.05). **d** IHC staining of SARS-CoV-2 N in all infected K18-hACE2 mice with or without LNA ASO treatment. Scale bar = 2 mm. The staining was repeated in lung collected from two independent batches of mice. **e** Expression changes of SARS-CoV-2 infection-upregulated genes in Saline- and LNA ASO-treated groups. Columns represent samples and rows represent genes. Colors indicate expression levels (log2 RPKM) relative to average expression across all samples. **f** Gene set enrichment analysis (GSEA) of Hallmark gene sets enriched in lungs of Saline- or LNA ASO-treated mice. Terms were ranked by the false discovery rate (q value). Source data are provided as a Source Data file.

Next, we examined the antiviral effect of 5’-ASO#26 by employing humanized transgenic K18-hACE2 mice, which are expressing human ACE2 allowing SARS-CoV-2 cell entry and infectious spread^25^. K18-hACE2 mice were inoculated with a high titer (1 × 10^4^ TCID_50_ units) of the USA-WA1/2020 SARS-CoV-2 strain via intranasal administration to allow consistent infection and phenotypic readouts in all animals. No significant weight loss was observed at 4 days post-infection (dpi) (Supplementary Fig. 5a), consistent with previous studies indicating that weight loss starts from 5 dpi^25^. Mice were treated with 5’-ASO#26 (400 µg) once-daily via intranasal administration in saline, from 3 days before infection until 3 dpi (Fig. 4a). High levels of infectious SARS-CoV-2 viral particles were detected in the lungs of the saline-treated control group (Saline), whereas a remarkable decrease (>80-fold) in the viral load in lungs was observed in the LNA ASO-treated group (Fig. 4b). Correspondingly, Remdesivir (Gilead), an approved treatment of COVID-19, also showed similar viral repressive effect in *Ces1c*^*−/−*^ mice (Supplementary Fig. 5b), which is a more proper model considering the low plasma stability of Remdesivir in wild-type mice^26,27^. As expected, the levels of viral *N* and *S* RNA were also potently (80–90-fold) repressed after LNA ASO treatment, as measured by RT-qPCR (Fig. 4c). We also demonstrated that 5’-ASO#26 repressed viral replication in vivo in a dose-dependent manner (Supplementary Fig. 5c). To further investigate the physiological effects of LNA ASO treatment in mice, histological analyses were carried out with control and LNA ASO-treated lung tissue after SARS-CoV-2 infection. The immunohistochemistry (IHC) analyses revealed clear repression of Nucleocapsid expression with LNA ASO treatment (Fig. 4d). Meanwhile, we did not observe significant histological changes by H&E staining in either Saline or LNA ASO-treated mice (Supplementary Fig. 5d), which is consistent with previous studies demonstrating that significant inflammation and severe alveolar wall thickening could only be observed at 7 dpi^25^. However, we did notice that the localized signal of Nucleocapsid correlated with local enrichment of CD3 (T cell marker) and B220 (B cell marker) by IHC, and moderate thickening of the alveolar wall (by H&E staining) in Saline-treated mice, but not in LNA ASO-treated mice (Supplementary Fig. 5d). The viral repressive effect of the LNA ASO was also examined by RNA-seq of lung tissues from mice with saline or LNA ASO treatment. Consistent with results from the TCID_50_ assay, viral RNA reads were dramatically decreased in LNA ASO-treated mice when compared with that of the Saline control (Supplementary Fig. 5e). To evaluate the in vivo effect of 5’-ASO#26, previously published RNA-seq data from infected K18-ACE2 mice^25^ were used to define up- and down-regulated genes in response to SARS-CoV-2 infection at 4 dpi (Supplementary Fig. 5i). As expected, expression profile changes induced by infection were markedly rescued by LNA ASO treatment (Fig. 4e and Supplementary Fig. 5f). Gene set enrichment analysis (GSEA) revealed strong enrichment of gene involved in type I and II interferon (IFN) signaling in infected mice treated with Saline when compared with 5’-ASO#26-treated group (Fig. 4f and Supplementary Fig. 5g), which is consistent with previous studies showing induction of antiviral defenses in response to SARS-CoV-2 infection^25^ and further proved that LNA ASO treatment did not trigger off-target inflammation in lung. Interestingly, the cholesterol homeostasis gene pathway was enriched in LNA ASO-treated mice (Fig. 4f and Supplementary Fig. 5h). It has been reported that the cholesterol biosynthesis pathway is necessary for SARS-CoV-2 infection^28,29^ and decreased levels of total cholesterol, high-density lipoprotein (HDL), and low-density lipoprotein (LDL) were reported in COVID-19 patients^30^, indicating a comprehensive role of intracellular and extracellular cholesterol during SARS-CoV-2 infection. The restored cholesterol homeostasis in the lungs of LNA ASO-treated mice further indicates the efficacy of the LNA ASO treatment.

### 5′-ASO#26 efficiently represses SARS-CoV-2 variants both in vitro and in vivo

**Fig. 5:**
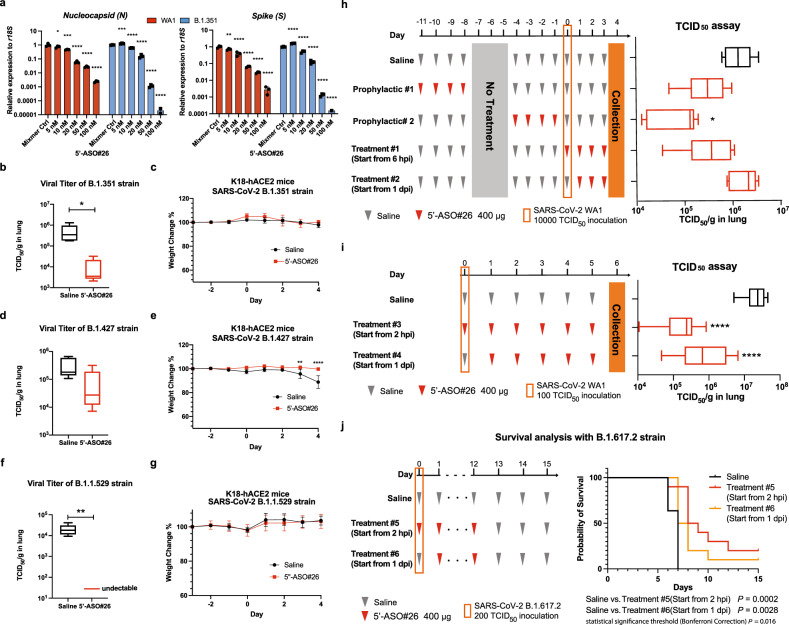
Prophylaxis and treatment of SARS-CoV-2 variant strains with 5′-ASO#26. **a** Dose-dependent effects of 5′-ASO#26 on inhibition of viral replication of SARS-CoV-2 WA1 and B.1.351 strains were evaluated in infected Huh-7 cells by RT-qPCR of Nucleocapsid protein (N) and Spike (S) RNA (*N* = 3, symbols represent mean ± SD). One-way ANOVA with Dunnett’s test was used to determine significance. For WA1 (Nucleocapsid), Mixmer Ctrl vs. 5 nM, * *P* = 0.0496; Mixmer Ctrl vs. 10 nM, *** *P* = 0.0003. For B.1.351 (Nucleocapsid), Mixmer Ctrl vs. 5 nM, ***P = 0.0001. For WA1(Spike), Mixmer Ctrl vs. 5 nM, **P = 0.0033 and groups marked with **** are *P* < 0.0001. **b**, **d** and **f** The viral burden in lungs of mice treated with Saline (*N* = 5) and 5′-ASO#26 (*N* = 5) was measured by TCID_50_ assay using lung homogenates. Student *t*-test was used to determine significance. In **b** *P = 0.0394 **c** and in **f**, ** *P* = 0.0060. **c**, **e** and **g** Weight change of mice in **b**, **d** and **f** was monitored (*N* = 5 for control or ASO-treated group in each experiment, symbols represent mean ± SD). Two-way ANOVA was used to determine significance, in **e** ** *P* = 0.0027, ****P < 0.0001. **h** Mice were administered with different treatment regimens of 5′-ASO#26 as indicated. Treatment on the day of infection was carried out at 6 hpi. The viral burden in lungs of mice in each group (*N* = 5) was measured by TCID_50_ assay using lung homogenates. One-way ANOVA with Dunnett’s test was used to determine significance (*P = 0.0293). **i** Mice were administered with different treatment regimens of 5′-ASO#26 as indicated. Treatment on the day of infection was carried out at 2 hpi. The viral burden in lungs of mice in each group (*N* = 7) was measured by TCID_50_ assay using lung homogenates. One-way ANOVA with Dunnett’s test was used to determine significance (****P < 0.0001). **j** Survival graph of mice administered with different treatment regimens of 5′-ASO#26 as indicated. Each group contain 5 male and 5 female K18-hACE2 mice. Log-rank (Mantel-Cox) test was used to determine significance and p value threshold was corrected by Bonferroni Correction. For **b**, **d**, **f**, **h** and **i**, center line, median; box limits, upper and lower quartiles; plot limits, maximum and minimum in the boxplot. Source data are provided as a Source Data file.

Because the 5′ leader sequence of SARS-CoV-2 is highly conserved and as 5′-ASO#26 does not target Spike, we predicted that 5′-ASO#26 should also be able to repress the replication of SARS-CoV-2 variant strains. Therefore, we tested several reported variants with 5′-ASO#26 in cell-based assays. Our results showed that 5’-ASO#26 exhibits potent repressive activity on viral replication (as assessed by *N* and *S* RT-qPCR) of multiple SARS-CoV-2 variants, including B.1.1.7 (Alpha), B.1.351 (Beta), P.1 (Gamma), B.1.427/B.1.429 (Epsilon), B.1.617.2 (Delta), B.1.1.529 (Omicron) and D614G (Fig. 5a and Supplementary Fig. 6a). We further tested the effect of 5′-ASO#26 in vivo to repress replication of the B.1.427, B.1.1.7, B.1.1.529 and B.1.617.2 strains in K18-hACE2 mice following the same schedule as shown in Fig. 4a. When testing the antiviral effect of 5′-ASO#26 with B.1.427 and B.1.351, we performed both TCID_50_ assays and RT-qPCR of lung homogenates to assess viral titer and observed that 5′-ASO#26 can also block viral replication of both variant strains in vivo (Fig. 5b, d; Supplementary Fig. 6b). Although mice were inoculated with the same amounts of variant strains, the in vivo replication rate of B.1.427 was significantly lower than that of B.1.351, however, the viral titers of both variant strains were repressed to a similar degree by LNA ASO treatment (Fig. 5b, d). Of note, similar to animals infected with the WA1 strain, the B.1.351 strain did not induce weight loss at 4 dpi (Fig. 5c). However, we noticed that there was a significant weight loss induced by B.1.427 strain infection from 3 dpi in the saline-treated group (Fig. 5e). In contrast, the LNA ASO-treated mice did not exhibit significant weight loss after B.1.427 strain infection (Fig. 5e), indicating that the viral repressive effect of LNA ASO treatment was still able to prevent the progression of SARS-CoV-2-induced pathologies. B.1.1.529 (Omicron) is the most recently emerged variant, and is highly contagious and has rapidly spread worldwide, outcompeting B.1.617.2 (Delta)^31^. Although computer modeling and in vitro binding data suggest that the B.1.1.529 Spike protein has higher affinity for the murine Ace2 receptor^32,33^, mice and hamsters showed an attenuation of infection^34^. As expected, 5′-ASO#26 treatment decreased the viral load of B.1.1.529 to nearly undetectable levels in the lungs of K18-hACE2 mice when compared with control treatment (Fig. 5f), and infection with B.1.1.529 (Omicron) did not induce significant weight loss in the control group (Fig. 5g).

To ensure the on-target effect of 5′-ASO#26, we used a mixmer LNA ASO control (Supplementary Table 3) alongside 5′-ASO#26 in mice infected with B.1.617.2 (Delta). While 5’-ASO#26 repressed viral replication in the lungs of K18-hACE2 mice infected with B.1.617.2 (Delta), mixmer LNA ASO control treatment did not show apparent effects on viral load, suggesting a lack of non-specific antiviral effects of the control LNA ASO (Supplementary Fig. 6c). Similar to the B.1.351 (Beta) strain or B.1.1.529 (Omicron), B.1.617.2 (Delta) also did not induce significant weight loss during infection in mice (Supplementary Fig. 6d).

### Administration of 5’-ASO#26 has both prophylactic and treatment effects

To explore the optimal treatment time course, we assessed the efficacy of 5′-ASO#26 viral repression in pre-infection and post-infection treatment regimens (Fig. 5h). We found that the strongest viral repressive effect was observed in the Prophylactic #2 group (Fig. 5h), in which the mice were treated with LNA ASO for four days, followed by infection 24 h later. Viral repression was also validated by IHC analysis of N in mouse lung tissues (Supplementary Fig. 6e). Notably, a prophylactic effect was still observed one week after LNA ASO treatment (Prophylactic #1, Fig. 5h). Although a previous study reported that intratracheal administration of a gapmer LNA ASO caused the predominant accumulation of LNA ASO in macrophages^22^, the prophylactic antiviral effect of LNA ASO treatment (Prophylactic #1) indicates that 5′-ASO#26 accumulates in lung epithelial cells. Starting LNA ASO treatment at 6 hpi also revealed a moderate repressive effect, whereas beginning treatment from 1 dpi showed no repressive effect in mice (Treatment #1 and #2, Fig. 5f). These results show that 5′-ASO#26 exhibits potent effects as a prophylactic therapeutic. The diminished ability of post-infection treatment of 5′-ASO#26 to inhibit viral replication may be a result of rapidly accumulating sub-genomic viral RNAs saturating the amount of administered LNA ASO in the lung due to the very large dose of virus used to inoculate the mice (10,000 TCID_50_ units). To approximate a lower virus exposure more accurately reflecting likely human exposure levels, we evaluated treatment response with mice inoculated with 100-fold lower (100 TCID_50_ units) infectious dose of the original WA1 strain, and our results show that 5′-ASO#26 significantly lowered viral load when treatment was initiated both at 2 hpi (Treatment #3) and 24 hpi (Treatment #4) (Fig. 5i). Next, we evaluated whether the ASO treatment would improve survival in vivo. We started the intranasal administration of 5′-ASO#26 from 2 hpi (Treatment #5) or 24 hpi (Treatment #6) until 12 days after infection (Fig. 5j) in K18-hACE2 mice with infection of B.1.617.2 (Delta) strain (200 TCID_50_ units). Although infection-induced weight loss was observed in all groups (Supplementary Fig. 6f), the survival period was improved both in Treatment #5 or Treatment #6 groups when compared with Saline control (Fig. 5j), and mice that survived in treatment groups fully regained their weight at the endpoint (15 dpi) (Supplementary Fig. 6f). A recent study reported transmission from pet hamsters to humans^35^. Therefore, we evaluated whether 5′-ASO#26 might block the viral airborne transmission by considering hamsters as a transmission model. Hamsters were first infected with B.1.1.529 (Omicron) strain (100 TCID_50_ units), and, surprisingly the administration of 5′-ASO#26 beginning from 1 dpi fully repressed the viral amplification in hamster lung to undetectable level (Supplementary Fig. 6g). We then tested the WA1 strain (100 TCID_50_ units) infection in hamsters. While the administration of 5′-ASO#26 starting from 1 dpi was unable to efficiently suppress viral replication in hamster lung (Supplementary Fig. 6h), analysis of nasal wash from these hamsters indicated a significant decrease of viral load in the nasal cavity after 5′-ASO#26 treatment (Supplementary Fig. 6i), suggesting that 5′-ASO#26 treatment may block airborne transmission of the virus. Taken together, these results demonstrate that our intranasal LNA ASO may be efficacious as either prophylactic or post-infection treatment regimens. Considering that we directly administered the naked 5′-ASO#26 intranasally in saline, we believe that additional modifications of the LNA ASO (such as lipid-conjugation^36–38^) may further promote cellular uptake of LNA ASO in lung, improving the effect of post-infection LNA ASO treatment. Direct delivery of the LNA ASO to the lung via inhaler or nebulizer may also improve post-infection therapeutic effect.

## Discussion

Antisense therapy is currently used in clinical treatment for a range of different diseases, including cytomegalovirus retinitis (Fomivirsen^39^), Duchenne muscular dystrophy (Eteplirsen^40^), and Spinal Muscular Atrophy (Nusinersen)^41^. Recently, an siRNA-based SARS-CoV-2 therapy was reported^42^. However, the siRNA targeting to SARS-CoV-2 required packaging with lipid nanoparticles (LNPs) that necessitates additional formulation and manufacturing steps and present the risk of off-target inflammatory responses. Moreover, it required invasive systemic delivery via intravenous administration^42^. Here we show that intranasally administered LNA ASOs represent a promising therapeutic strategy for virus-induced respiratory diseases such as COVID-19. Importantly, our study demonstrated that naked LNA ASOs delivered intranasally in saline exhibit potent efficacy in vivo and were well tolerated, and no formulation is necessary to achieve both prophylactic and therapeutic effect. Considering the relatively small-sized genomes of RNA viruses and the ability to rapidly determine the sequence of any viral genome by next-generation sequencing (NGS) techniques, the design and screening of antiviral LNA ASOs can be fast and efficient, allowing for a rapid response to other global health crises posed by emerging viral threats in the future. The apparent ability of single-stranded RNA viruses to accumulate immune-evading mutations present great challenges for vaccine and therapeutics development. Of note, LNA ASOs can overcome the challenge of mutations due to the ability to design sequences flexibly and specifically targeted to highly conserved and critical regulatory regions of the viral genome. Several potential antibody-based^43^ or inhibitory peptide-based strategies^44,45^ for nasal prophylactics and therapeutics targeting SARS-CoV-2 have very recently been developed, however, they either target the Spike protein, which is frequently mutated and may thus escape inactivation, or they target a human host protein (e.g. TMPRSS2), with potentially unknown deleterious consequences. Similarly, small molecule compounds identified using traditional drug screening approaches^46^ cannot conveniently be modified or adjusted for the emergence of new mutated variants that circumvent inhibition. Combinatorial LNA ASO cocktails targeting multiple essential genomic regions of viruses may further increase the efficacy of LNA ASOs as therapeutic candidates to overcome viral evasion mutations and a nasal delivery of LNA ASO combined with antiviral-related cytokines like interferon-λ^47^ may further improve the clinical performance of LNA ASO therapy. In conclusion, we have identified an intranasally delivered LNA ASO targeting the 5’ leader sequence as a viable therapeutic approach for preventing or treating SARS-CoV-2 infections, including those caused by variants of concern, suggesting that LNA ASOs represent attractive candidates for the treatment of COVID-19.

## Methods

### Cell culture and viruses

Huh-7 and Vero E6 cells were cultured in Dulbecco’s Modified Eagle Medium (DMEM), Vero E6-TMPRSS2-T2A-ACE2 was cultured in DMEM with 10 μg/ml puromycin (Sigma) and Vero 81 cells were cultured in MEM α (Minimum Essential Medium α). The media were supplemented with 10% fetal bovine serum (FBS) and 1% Penicillin/Streptomycin. Cells were maintained at 37 °C in 5% CO_2_. Transfection of Huh-7 cells was performed with Lipofectamine 3000 reagent (ThermoFisher). Cells were infected by SARS-CoV-2 virus 12 hrs after transfection. The 2019n-CoV/USA_WA1/2020 isolate of SARS-CoV-2 was obtained from the US Centers for Disease Control and Prevention. Infectious stocks were produced by inoculating Vero E6 cells and collecting the cell culture media upon observation of cytopathic effect; debris were removed by centrifugation and passage through a 0.22 μm filter. The supernatant was then aliquoted and stored at −80 °C. D614G, B.1.427, B.1.1.7 and B.1.617.2 strains were kind gifts from Dr. Mary Kate Morris at the California Department of Public Health (CDPH).

### Locked nucleic acid antisense oligonucleotides (LNA ASOs)

ASOs targeting the SARS-CoV-2 5′ leader sequence, downstream sequences in the 5′ untranslated region (UTR) of ORF1a/b, and ORF1a/b FSE were designed by sliding a window of 15 to 17 nt across the aforementioned target sequences (Supplementary Table 1). Mixmers were designed with 2–3 LNA modifications at the 5′ and 3′ ends and 5–6 additional LNA modifications interspersed across the DNA-PS (Phosphorothioate Bond) sequence aimed at maximizing binding affinity with minimal self-complementarity. Gapmers were designed using the same target recognition sequences as mixmers, but with 3–4 LNA modifications at the 5′ and 3′ ends of the gapmer ASO (LNA wings) and a DNA-PS window of 9–10 nucleotides enabling recruitment of RNase H for SARS-CoV-2 target RNA cleavage. LNA ASOs were purchased from Integrated DNA Technologies (IDT). For the screening, small scale synthesis (100 nmole) of all LNA ASOs was followed by standard desalting purification. For the animal trials, large scale synthesis (250 mg) of the 5′-ASO#26 LNA ASO was followed by HPLC purification and endotoxin test. Control LNA ASOs are listed in Supplementary Table 3.

### Biosafety

All aspects of this study were approved by the office of Environmental Health and Safety at UC Berkeley before initiation of this study. Work with SARS-CoV-2 was performed in a biosafety level 3 laboratory by personnel equipped with powered air-purifying respirators.

### Animals

C57BL/6J, C57BL/6J *Ces1c*^*−/−*^ [B6.Cg-Ces1c^tm1.1Loc^/J] and K18-hACE2 [B6.Cg-Tg(K18-ACE2)2Prlmn/J] mice were purchased from the Jackson Laboratory and male golden Syrian hamsters at 8 weeks old were obtained from Charles River Labs (Strain Code:049). For testing LNA ASO, 8 to10 weeks old K18-hACE2 mice (male or female) and 9 weeks old hamsters (male) were anesthetized using isoflurane and intranasally inoculated with 1 × 10^4^ TCID_50_ and 100 TCID_50_ of SARS-CoV-2, respectively. For the treatment in mice, saline (40 µl) or 5′-ASO#26 (indicated amount of LNA ASO in 40 µl saline) was administered once-daily intranasally and in hamsters, saline (60 µl) or 5′-ASO#26 (indicated amount of LNA ASO) was administered once-daily intranasally. Mice and hamsters were sacrificed at indicated days post infection (dpi). For testing Remdesivir (TargetMOI), C57BL/6J *Ces1c*^*−/−*^ mice were anesthetized using isoflurane and inoculated with 2 × 10^4^ TCID_50_ of SARS-CoV-2 MA10 strain intranasally. For the treatment, C57BL/6J *Ces1c*^*−/−*^ mice were administrated twice daily (B.I.D.) with Remdesvir (RDV; 10 mg/kg or 25 mg/kg) or Vehicle (Veh: 12% w/v sulfobultylether-beta-cyclodextrin and 2% DMSO in water, pH 5.0) subcutaneously. For all infected mice, the left lung lobe was collected and lysed for fifty-percent tissue culture infective dose (TCID_50_) assay, the inferior lobe was collected for RNA extraction and the post-caval lobe was collected for histological analysis, Immunofluorescence (IF) staining or fluorescent in situ hybridization (FISH) analysis.

### Nasal wash in hamsters

Hamsters were anesthetized using isoflurane and sacrificed for nasal wash collection. The nasal wash samples were collected by making an incision in the trachea and 2 ml of DMEM medium was slowly injected into nasal cavity by using a blunt cannula monoject (McKesson) and injected medium was collected from nostrils (~1.8 ml). The nasal wash samples were then subjected to DNA/RNA shield reagent (Zymo Research) for RNA extraction.

### Ethics

All procedures involving the use of mice and hamsters were approved by the University of California, Berkeley Institutional Animal Care and Use Committee (Protocol # AUP-2018-10-11513-1). All protocols conform to federal regulations, the National Research Council Guide for the Care and Use of Laboratory Animals, and the Public Health Service Policy on Humane Care and Use of Laboratory Animals.

### Fifty-percent tissue culture infective dose (TCID_50_) assay

Virus viability and titers were evaluated in TCID_50_ assay within Vero E6 cells for most SARS-CoV-2 variants, TCID_50_ assay of B.1.617.2 was carried out within Vero 81 and TCID_50_ assay of B.1.1.529 was carried out within Vero E6-TMPRSS2-T2A-ACE2. Briefly, ten thousand cells were plated in each well in 96-well plates and cultured at 37 °C overnight. Medium from SARS-CoV-2-infected cells or lysates from mouse lungs were used for ten-fold serial dilution with DMEM and added to the 96-well plates of Vero E6 cells. The plates were observed for cytopathic effect (CPE) after 3 days of culturing. The TCID_50_ results were calculated using the Spearman and Karber method^48^.

### RNA extraction and real-time quantitative PCR (RT-qPCR)

Infected Huh-7 cells (with or without medium) or mouse lung tissues were lysed in DNA/RNA shield reagent (Zymo Research) and total RNA was extracted by using RNeasy kit (Qiagen) according to the manufacturer’s protocol. cDNA was prepared by using 100 ng total RNA with iScript™ Reverse Transcription Supermix (BioRAD) and qPCR was performed with Fast SYBR™ Green Master Mix (ThermoFisher) and the reaction was run on the QuantStudio6 System under defaulted Fast Mode (denaturation: 95 °C/1 s, annealing/extension: 60 °C/20s, 40 cycles) (Applied Biosystems). mRNA levels of viral *Nucleocapsid* and *Spike* were normalized to that of human or mouse r18S, respectively. Each primer in the reaction was diluted to 0.25 µM and the qPCR primer sets (purchased from IDT) are listed in Supplementary Table 3.

### RNA-sequencing

cDNA libraries were constructed from 500 ng of total RNA from Huh-7 or lung tissues of mice according to the manufacturer’s protocol of Stranded mRNA-seq kit (KAPA). Briefly, mRNA was captured and fragmentized to 100–300 bp. library construction was performed undergoing end repair, A tailing, ligation of unique dual-indexed adapters (KAPA) and amplification of 10 cycles to incorporate unique dual index sequences. Libraries were sequenced on the NovaSeq 6000 (Novogene) targeting 40 million read pairs and extending 150 cycles with paired end reads. STAR aligner^49^ was used to map sequencing reads to transcripts in the mouse mm10 reference genome. Read counts for individual transcripts were produced with HTSeq-count^50^, followed by the estimation of expression values and detection of differentially expressed transcripts using EdgeR^51^. Differentially expressed genes were defined by at least 2-fold change with FDR less than 0.01.

### DMS modification of in vitro-transcribed RNA

gBlock containing the first 3,000 nucleotides of the SARS-CoV-2 genome (2019-nCoV/USA-WA1/2020) was obtained from IDT. The gBlock was amplified by PCR with a forward primer that contained the T7 promoter sequence gBlock-F and the reverse primer gBlock-R listed in Supplementary Table 3. The PCR product was used as the template for T7 Megascript in vitro transcription (ThermoFisher Scientific) according to manufacturer’s instructions. Next, 1 µl of Turbo DNase I (ThermoFisher Scientific) was added and incubated at 37 °C for 15 min. The RNA was purified using RNA Clean and Concentrator^TM^-5 (Zymo). Between 1 and 2 µg RNA was denatured at 95 °C for 1 min. Denatured RNA was refolded in the presence of 2 µM of LNA ASO by incubating the mixture in 340 mM sodium cacodylate buffer (Electron Microscopy Sciences) and 5 mM MgCl^2+^, such that the volume was 97.5 µl, for 20 min at 37 °C. Then, 2.5% DMS (Millipore-Sigma) was added and incubated for 5 min at 37 °C whole shaking at 800 r.p.m. on a thermomixer. Subsequently, DMS was neutralized by adding 60 µl β-mercaptoethanol (Millipore-Sigma). The RNA was precipitated by incubating in 3 µl (45 µg) glycoblue coprecipitant (Invitrogen), 18 µl 3M sodium acetate and 700 µl ethanol between 1 h and overnight at −80 °C, followed by centrifugation at max speed for 45 min in 4 °C. The RNA was washed with 700 µl ice cold 75% ethanol and centrifuged for 5 min. RNA was resuspended in 10 µl water.

### DMS modification of infected Huh-7 cells with ASO treatment

Huh-7 cells were transfected with LNA ASO (50 nM) 12 h before the infection. After infection of SARS-CoV-2 (MOI 0.05), cells were cultured in 6-well plates with 2 ml of media. Then, 2.5% DMS was added to cells and incubated for 3 min at 37 °C. Subsequently, after careful removal of the media, DMS was neutralized by adding 20 ml of chilled 10% β-mercaptoethanol in PBS. The cell pellets were washed once with chilled PBS and collected for RNA extraction.

### rRNA subtraction of total cellular RNA from DMS-treated cells

Between 3 and 5 µg RNA per sample was used as the input for rRNA subtraction. First, equal amount of rRNA pooled oligonucleotides were added and incubated in hybridization buffer (200 mM NaCl, 100 mM Tris-HCl, pH 7.4) in a final volume of 60 µl. The samples were denatured for 2 min at 95 °C, followed by a reduction of 0.1 °C/s until the reaction reached 45 °C. A total of 3–5 µl Hybridase^TM^ Thermostable RNase H (Lucigen) and 7 µl 10× RNase H buffer preheated to 45 °C was added. The samples were incubated at 45 °C for 30 min. The RNA was purified using RNA Clean and Concentrator^TM^-5 kit and eluted in 42 µl water. Then, 5 µl Turbo DNase buffer and 3 µl Turbo DNase (ThermoFisher Scientific) were added to each sample and incubated for 30 min at 37 °C. The RNA was purified using RNA Clean and Concentrator^TM^-5 kit and eluted in 10 µl water.

### RT-PCR and sequencing of DMS-modified RNA

To reverse transcribe, rRNA-depleted total RNA or in vitro-transcribed RNA purified from the previous steps was added to 4 µl 5× FS buffer, 1 µl dNTP, 1 µl of 0.1 M DTT, 1 µl RNase Out, 1 µl of 10 µM reverse primer (5′-TCGTTGAAACCAGGGACAAG-3′, purchased from IDT) and 1 µl TGIRT-III (Ingex). The reaction was incubated for 1.5 h at 60 °C. Then, to degrade the RNA, 1 µl of 4 M NaOH was added and incubated for 3 min at 95 °C. The cDNA was purified in 10 µl water using the Oligo Clean and Concentrator^TM^ kit (Zymo). Next, 1 µl of cDNA was amplified using Advantage HF 2 DNA polymerase (Takara) for 25–30 cycles according to the manufacturer’s instructions with 5′-leader primer set listed in Supplementary Table 3. The PCR product was purified using E-Gel^TM^ SizeSelect^TM^ II 2% agarose gel (Invitrogen). RNA-seq library for 300 bp insert size was constructed following the manufacturer’s instructions (NEBNext Ultra^TM^ II DNA Library Prep Kit). The library was loaded on iSeq-100 Sequencing flow cell with iSeq-100 High-throughput sequencing kit and library was run on iSeq-100 (paired-end run, 151 × 151 cycles).

### Immunohistochemistry (IHC) and hematoxylin and eosin (H&E) staining

Histology was performed by HistoWiz Inc. (histowiz.com) using a Standard Operating Procedure and fully automated workflow. Samples were processed, embedded in paraffin, and sectioned at 4 μm. Immunohistochemistry was performed on a Bond Rx autostainer (Leica Biosystems) with enzyme treatment (1:1000) using standard protocols. Antibodies used were rabbit monoclonal CD3 primary antibody (Abcam, ab16669, 1:100), rabbit monoclonal B220 primary antibody (Novus, NB100-77420, 1:10000), rabbit monoclonal SARS-CoV-2 (COVID-19) nucleocapsid primary antibody (GeneTex, GTX635686, 1:8000). Bond Polymer Refine Detection (Leica Biosystems) was used according to the manufacturer's protocol. After staining, sections were dehydrated and film coverslipped using a TissueTek-Prisma and Coverslipper (Sakura). Whole slide scanning (40×) was performed on an Aperio AT2 (Leica Biosystems).

### Analysis of bronchoalveolar lavage fluid (BALF) from LNA ASO-treated mice

Wildtype mice were treated intranasally with saline (40 µl) or 5′-ASO#26 (400 µg in 40 µl saline) once-daily for three days. Mice treated intranasally with lipopolysaccharides (60 µg in 40 µl saline, LPS from Escherichia coli O111:B4, Sigma) for one day were used as positive control. One day after the last administration, mice were sacrificed under anesthesia with 3% isoflurane, then BALF samples were collected by making an incision in the trachea and washing the lungs 5 times with 1 mL PBS, and repeating again with additional 1 ml PBS. BALF samples from each mouse were centrifuged at 450 g for 5 min at 4 °C and the supernatant was used for cytokine/chemokine measurement using the Bio-Plex MAGPix multiplex reader and Bio-Plex Pro Mouse Chemokine Panel 31-Plex panel (BioRad) following the manufacturer’s instructions. In addition, IFN alpha in BALF was measured with a Mouse IFN-alpha ELISA Kit (R&D system) following the manufacturer’s instructions.

### Flow cytometry analysis

Cells collected from BALF were incubated in 100 μl 1×RBC lysis buffer (BioLegend) on ice for 10 min, and then quenched by adding 1 ml PBS. Cells from each sample were resuspended in 100 μl Stain Buffer (554656, BD Pharmingen) and incubated for 15 min on ice with 1 μg mouse IgG (ab37355, Abcam, 1:500) for Fc blocking. Then cells were incubated with primary monoclonal antibodies including rat anti-mouse Gr1/Ly-6G-Alexa Fluor 594 (Novus, 1:200), rat anti-mouse CD11b-FITC (R&D System,1:200) and rat anti-F4/80-APC (R&D System,1:200). Flow cytometry was performed using FACS LSR Fortessa X20 (BD Biosciences) and data were analyzed by BD FACSDiva 8.0.1 and FlowJo v10.8.1.

### Immunofluorescence (IF) staining

Lung lobes were fixed with formalin solution (HT5014, Sigma) at room temperature for 5 min. Then the slides were blocked for 1 h at room temperature by 5% horse serum in 0.3% TritonX-100/PBS and then incubated overnight at 4 °C with the appropriate primary antibodies. The nuclei were stained with ProLong™ Gold Antifade Mountant with DAPI (ThermoFisher). The antibodies used for IF staining includes rat anti-mouse Gr1/Ly-6G-Alexa Fluor 594 (Novus, 1:200), rat anti-mouse CD11b-FITC (R&D System,1:200) and rat anti-F4/80-APC (R&D System,1:200). The sections were washed with PBS, dried at room temperature and observed via LSM710 confocal microscope (Zeiss).

### Fluorescent in situ hybridization (FISH) analysis

For analysis of the absorption and distribution of LNA ASO in administered mice, a FISH probe targeting to 5’-ASO#26 was designed and purchased from IDT (Supplementary Table 3). For FFPE tissue sections, slides were deparaffinated in Xylene and 100% ethanol and incubated in 1x Universal HIER antigen retrieval reagent (ab208572, Abcam) at 95 °C for 20 min followed by a wash step in 2xSSC buffer. For frozen tissue sections, slides were washed in PBS to remove OCT followed by fixation in fixative (3:1 methanol:acetic acid) for 5 min. Then tissue sections were digested in Pepsin solution (003009, ThermoFisher) at 37 °C for 30 min and dehydrated via 70%, 85% and 100% EtOH. The FISH probe (0.5 μM) was prepared in the hybridization solution (50% formamide, 10% dextran sulfate, 0.1% SDS, 300 ng/ml Salmon Sperm DNA in 2xSSC buffer). The slides incubated with FISH probe were desaturated at 75 °C (for FFPE sections) or 73 °C (for frozen sections) for 5 min and incubated at 37 °C for 16 h in HYBrite (VYSIS). Slides were washed in Wash Buffer 1 (0.3% NP-40 in 0.4× SSC buffer) at 73 °C for exactly 2 min, and then in Wash Buffer 2 (0.1% NP-40 in 2× SSC buffer) at room temperature for 1 min. The nuclei were stained with ProLong™ Gold Antifade Mountant with DAPI (ThermoFisher) and slides were observed via LSM710 confocal microscope (Zeiss).

### Statistics and reproducibility

Data are presented as mean values, and error bars represent SD. Data analysis was performed using GraphPad Prism 8. Data were analyzed using unpaired t-test; one-way or two-way ANOVA followed by Turkey or Dunnett test as indicated. All statistical analyses were two-sided and *P* value < 0.05 was considered as statistically significant. All in vitro experiments were repeated at least twice and for in vivo experiment and showed good consistency, all in vivo experiments conducted in C57BL/6J, K18-hACE2 mice and hamsters with SARS-CoV-2 WA1 and D.1.617.2 strain were repeated twice with good consistency and the other variants were only tested once in K18-hACE2 mice or hamsters. For Remdesivir, tests in C57BL/6J *Ces1c*^*−/−*^ mice were only tested once due to the limited number of animals.

### Reporting summary

Further information on research design is available in the Nature Research Reporting Summary linked to this article.

## Supplementary information

Supplementary Information

Reporting Summary

## Source data

Source Data

## Data Availability

All data are available in the manuscript and associated files. Source data is provided with this paper. The data have been deposited in NCBI’s Gene Expression Omnibus and are accessible through GEO Series accession number GSE174382. The published RNA-seq data of infected K18-hACE2 at 0 dpi and 4 dpi were obtained from GSE154104. Source data are provided with this paper.
