# Supplementary material for: An intranasal ASO therapeutic targeting SARS-CoV-2
Source: Nat Commun. 2022 Aug 3;13:4503. doi: 10.1038/s41467-022-32216-0 (PMC9349213; doi:10.1038/s41467-022-32216-0)
Supplement: Supplementary file 1 — Supplementary Information [file 41467_2022_32216_MOESM1_ESM.pdf]

Supplementary Information

Title: An intranasal ASO therapeutic targeting SARS-CoV-2

Supplementary Figures:

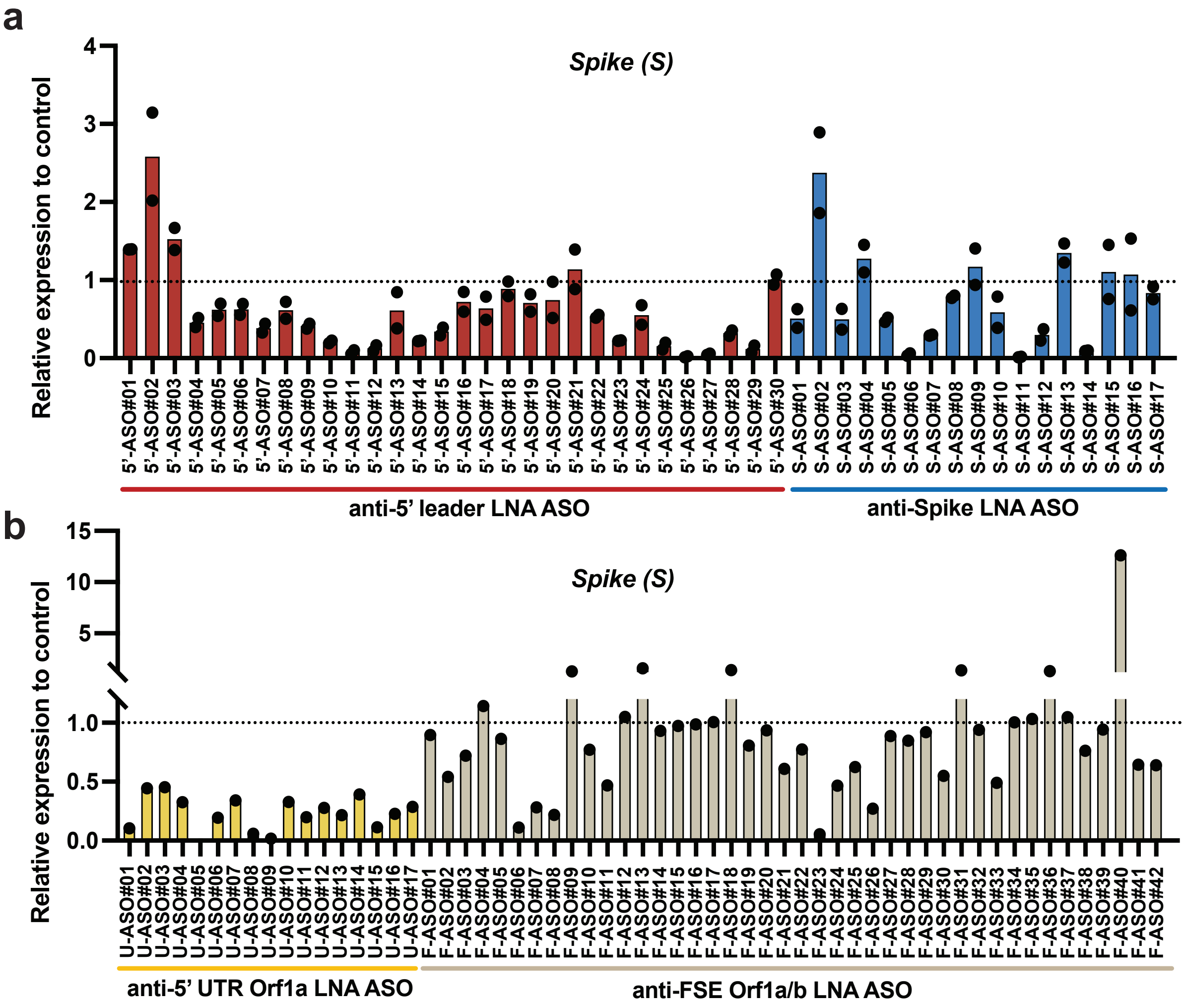

Supplementary Figure 1. *In vitro* screening of LNA ASOs targeting SARS-CoV-2.
a) and b) Infected Huh-7 cells were treated with LNA ASO (100 nM) and cell culture media was collected at 48 hpi. Levels of viral Spike (S) RNA were analyzed by RT-qPCR. Each LNA ASO was tested in duplicate and compared with in vitro Mixmer control LNA ASO or Gapmer control LNA ASO. One-way ANOVA with Dunnett's test was used to determine significance (**** $P < 0.0001$). Source data are provided as Source Data files.

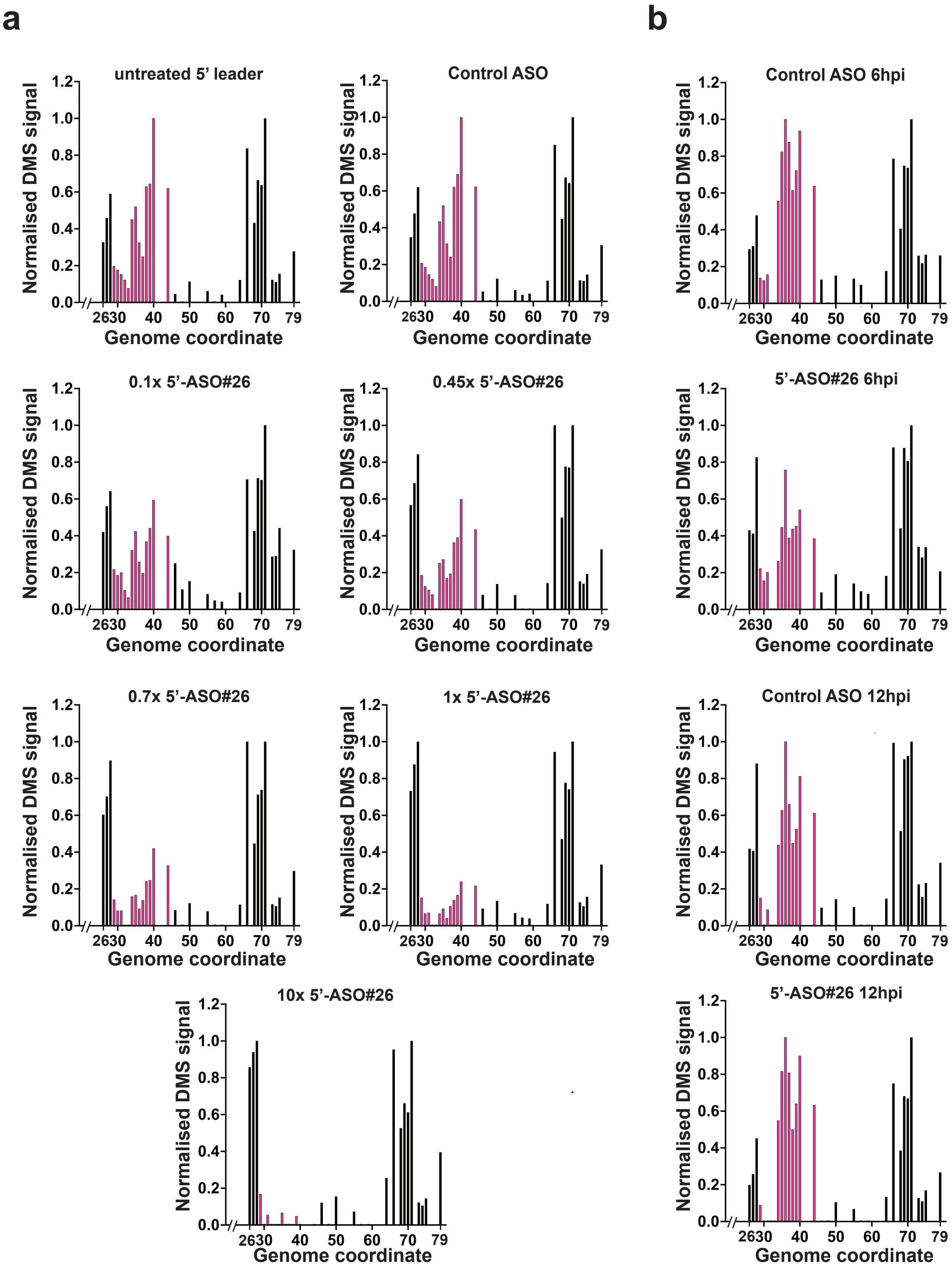
a
b
untreated 5' leader
Control ASO
Control ASO 6hpi
0.1x 5'-ASO#26
0.45x 5'-ASO#26
5'-ASO#26 6hpi
0.7x 5'-ASO#26
1x 5'-ASO#26
Control ASO 12hpi
10x 5'-ASO#26
5'-ASO#26 12hpi
Normalised DMS signal
Genome coordinate
0.0
0.2
0.4
0.6
0.8
1.0
1.2
26
30
40
50
60
70
79

Supplementary Figure 2. 5’-ASO#26 binding to the viral target at different stoichiometry *in vitro* and in Huh-7 cells.

a) Plots showing normalised DMS signals of *in vitro*-transcribed SARS-CoV-2 5’leader without addition of any ASO (top left), following the addition of control LNA ASO (top right), titration with 5’ -ASO#26 at 0.1x (second row, left), 0.45x (second row, right), 0.7x (third row, left), 1x (third row, right) and 10x (bottom row) molar ratio of 5’-ASO#26. b) Plots showing normalised DMS signals in Huh-7 cells transfected with control LNA ASO harvested 6 hpi (top), transfected with 5’ -ASO#26 harvested 6hpi (second row), transfected with control LNA ASO harvested 12 hpi (third row), and transfected with 5’ -ASO#26 harvested 12hpi (bottom row). For a) and b) only nucleotides from positions 26 to 79 in the SARS-CoV-2 genome are included. Each bar represents one nucleotide, and the 5’-ASO#26 target region is coloured in pink. Source data are provided as Source Data files.

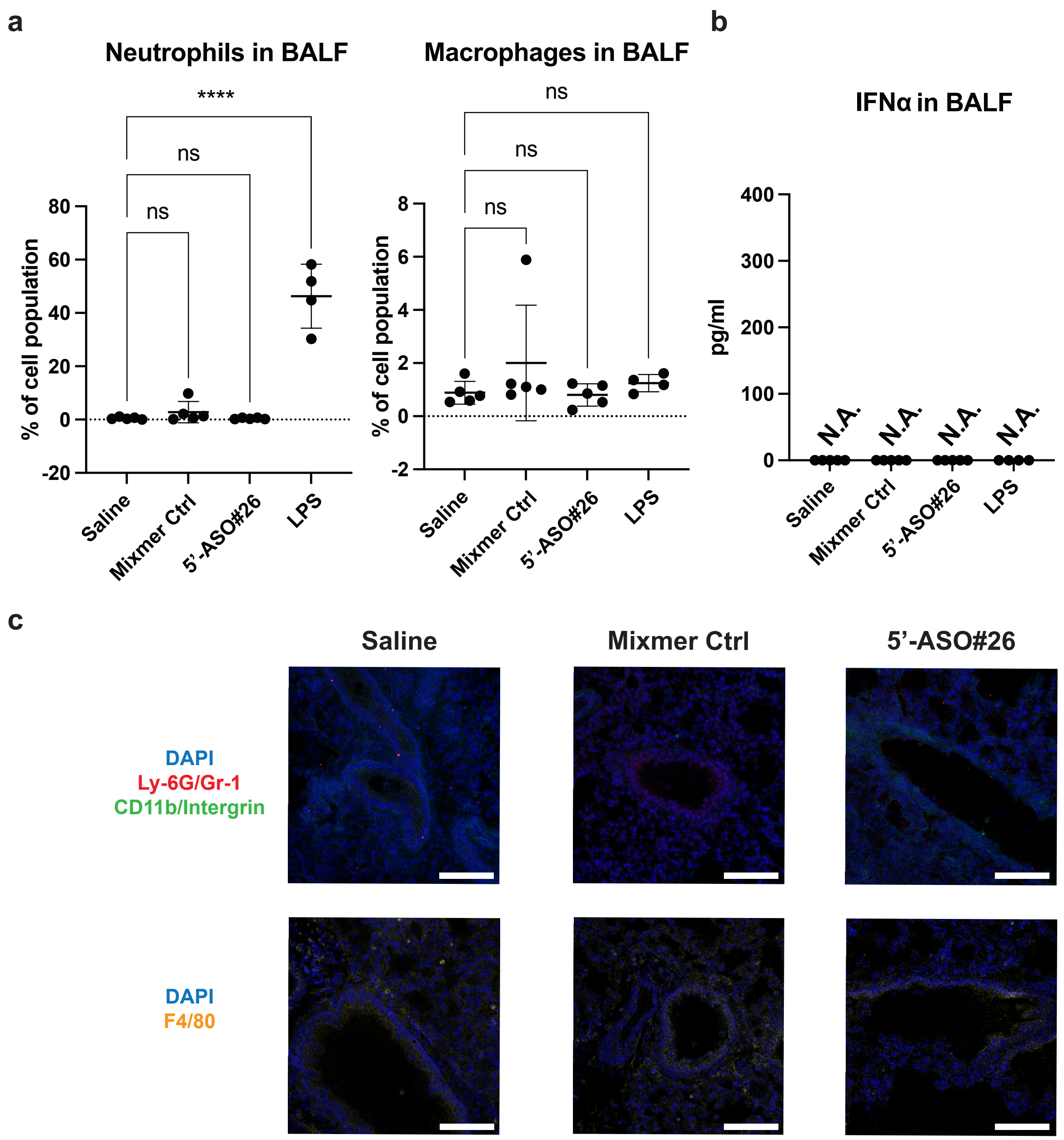

Supplementary Figure 3. Evaluation of lung delivery efficacy and potential immune-stimulatory effects of LNA ASOs.
a) The percentage of neutrophils and macrophages in BALF collected from mice with three days-treatment of LNA ASOs or Saline (N=5, symbols represent mean ± SD); one day-treatment of LPS was used as a positive control (N=4). b) INFα ELISA in BALF collected from mice with three days-treatment of LNA ASOs or Saline (N =5 for Saline, Mixmer Control and 5'-ASO#26 and N=4 for LPS group). c) Representative result of IF staining in lung collected from mice with three days-treatment of LNA ASOs or Saline. Scale bar = 100 μm. The IF staining was repeated twice. For A), one-way ANOVA with Dunnett's test was used to determine significance **** $P < 0.0001$ and ns, not significant. Source data are provided as Source Data files.

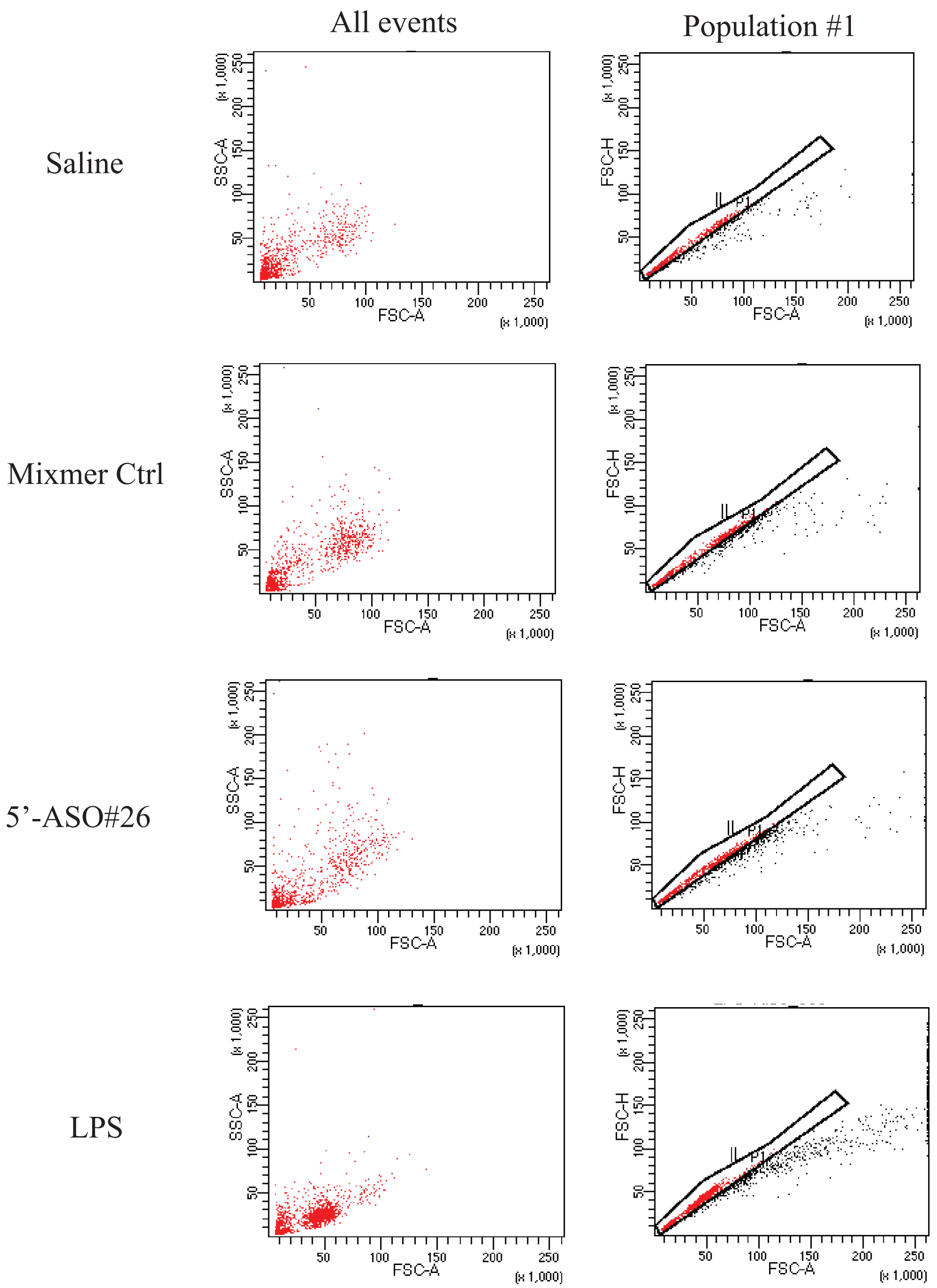

Supplementary Figure 4. Gating strategy for BALF cell analyzer.

All events were pre-gated by FSC and SSC to make sure >95% populations were visible in the scales and cell doublets were excluded by FSC-A/H. All four conditions (Saline, Mixmer Control, 5'-ASO#26 and LPS) are using the same gating strategy.

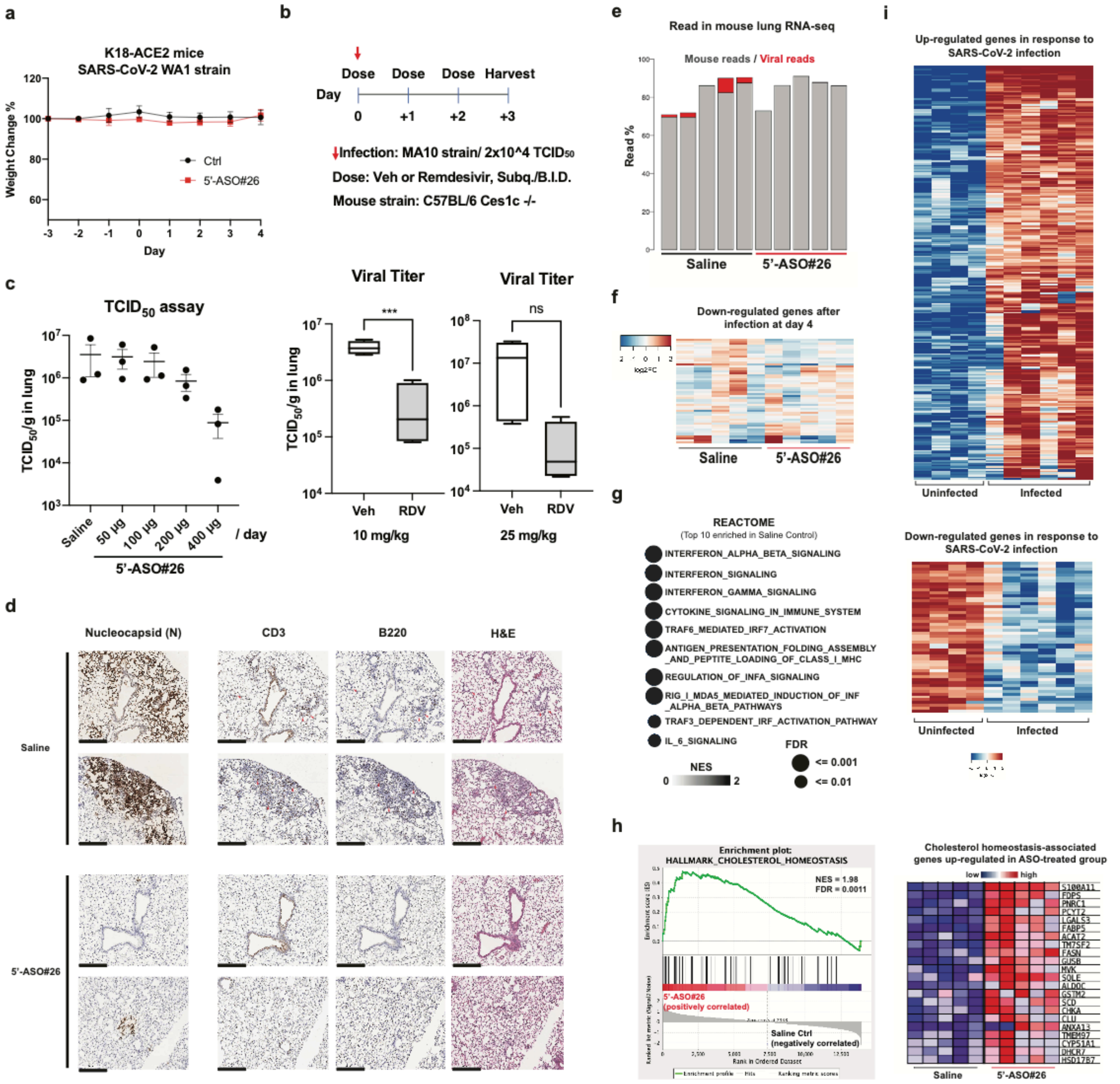

Supplementary Figure 5. Evaluating the *in vivo* effects of 5'-ASO#26 in K18-hACE2 mice. a) Weight change of mice was monitored over the treatment/infection course (N=5 in each group, symbols represent mean ± SD). b) Remdesivir efficacy testing in *Ces1c*$^{-/-}$ mice. Mice were administered with different doses of remdesivir as indicated. The viral burden in lungs of mice in each group (N=4) were measured by $TCID_{50}$ assay using lung homogenates. Student *t*-test was used to determine significance (*** $P = 0.0004$, two-sided, no adjustment for multiple comparison). Center line, median; box limits, upper and lower quartiles; plot limits, maximum and minimum in the boxplot. c) LNA ASO dose-dependence efficacy testing in mice. Mice were administered with different doses of 5'-ASO#26 in 40 µl saline as indicated. The viral burden in lungs of mice in each group (N=3, symbols represent mean ± SD) were measured by $TCID_{50}$ assay using lung homogenates. d) Representative images of hematoxylin and eosin (H&E) staining of lung sections

and immunohistochemistry (IHC) staining of CD3 and B220 in infected K18-hACE2 mice with Saline (N=5) or 5'-ASO#26 treatment (N=5). Scale bar = 500 µm. e) Percentage of mouse/viral sequencing reads in RNA-seq data of Saline- and LNA ASO-treated mouse lung. Reads mapped to the mouse genome are colored in gray and reads mapped to the virus are colored in red. f) Expression changes of SARS-CoV-2 infection-downregulated genes in Saline- and LNA ASO-treated groups. g) GSEA of REACTOME gene sets enriched among upregulated genes in lungs of Saline-treated mice. Terms were ranked by the false discovery rate (q value). h) GSEA plot and heatmap of significantly upregulated genes enriched in the cholesterol homeostasis pathway in LNA ASO-treated mice. i) Heatmaps of significantly upregulated and downregulated (> 2-fold, FDR<0.01) genes at day 4 after SARS-CoV-2 infection. For f), h) and i) Columns represent samples and rows represent genes. Colours indicate gene expression levels (log2 RPKM) relative to average expression across all samples. Source data are provided as Source Data files.

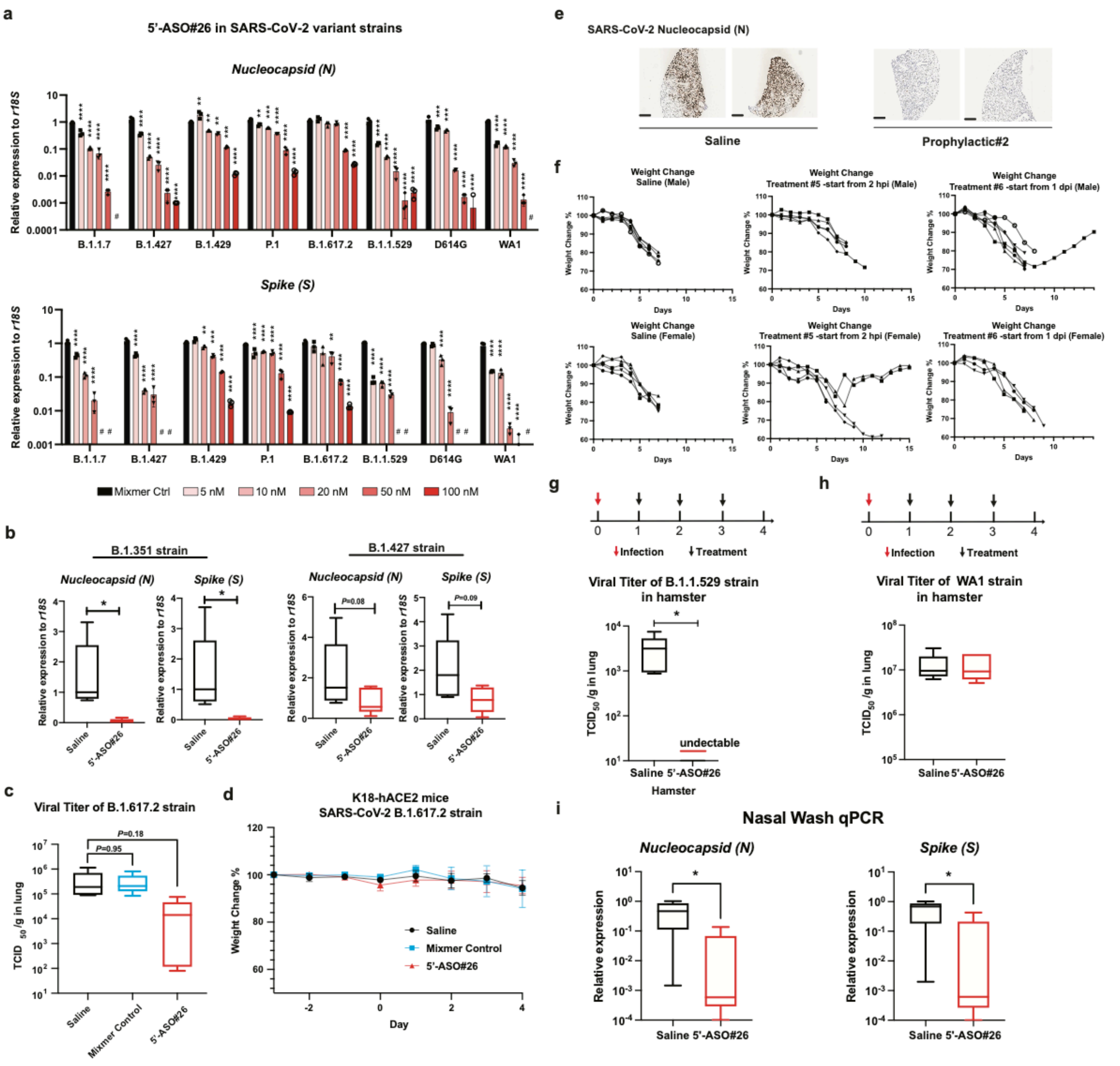

Supplementary Figure 6. Assessing the effects of 5'-ASO#26 in repressing the replication of SARS-CoV-2 variants.

a) Dose-dependent efficacy of 5'-ASO#26 in repressing replication of SARS-CoV-2 variant strains was evaluated in infected Huh-7 cells with increasing doses of 5'-ASO#26 by RT-qPCR of Nucleocapsid (N) and Spike (S) RNAs (N=3, symbols represent mean ± SD). For B.1.429 (Nucleocapsid), Mixmer Ctrl vs. 5 nM, ** P = 0.0034; Mixmer Ctrl vs. 10 nM, ** P = 0.0068; Mixmer Ctrl vs. 20 nM, ** P = 0.0015; Mixmer Ctrl vs. 50 nM, *** P = 0.0001. For P.1(Nucleocapsid), Mixmer Ctrl vs. 5 nM, ** P = 0.0034; Mixmer Ctrl vs. 10 nM, *** P = 0.0007. For D614G(Nucleocapsid), Mixmer Ctrl vs. 5 nM, *** P = 0.0008; Mixmer Ctrl vs. 10 nM, *** P = 0.0002. For B.1.429 (Spike), Mixmer Ctrl vs. 10 nM, ** P = 0.0087, Mixmer Ctrl vs. 20 nM, *** P = 0.0004. For B.1.617.2 (Spike), Mixmer Ctrl vs. 20 nM, ** P = 0.0058 and groups marked

with **** are $P < 0.0001$. b) B.1.427 and B.1.351 viral RNA levels in mouse lungs were analyzed by RT-qPCR of Nucleocapsid (N) and Spike (S) RNAs (N=5). For B.1.351, *P (Nucelocapsid) = 0.0152 and *P (Spike) = 0.0371. c) The B.1.617.2 viral burden in lungs of mice treated with Saline (N=5), Mixmer control ASO (N=5) and 5'-ASO#26 (N =5) was measured by $TCID_{50}$ assay using lung homogenates. d) Weight change of mice in c) was monitored (N=5, symbols represent mean ± SD). e) Representative images of IHC staining of SARS-CoV-2 Nucleocapsid protein in Saline (N=5) or Prophylactic#2 (N=5) regimen groups. Scale bar = 2 mm. f) Weight change of mice in survival test. g) and h) The B.1.1.529 and WA1 viral burden in lungs of hamsters treated with Saline (N=5) and 5'-ASO#26 (N =5) was measured by $TCID_{50}$ assay using lung homogenates collected at 4 dpi. Hamsters were inoculated with 100 $TCID_{50}$ units of virus and the treatment (Saline or 2 mg 5'-ASO#26/day) started from 1 dpi to 3 dpi. * P = 0.0308 i) Nasal wash qPCR for hamsters in h). Viral RNA levels in hamster nasal wash were analyzed by RT-qPCR of Nucleocapsid (N) (*P = 0.0354) and Spike (S) (*P = 0.0404) RNAs. For a) and c), One-way ANOVA with Dunnett's test was used to determine significance (** $P<0.01$, *** $P < 0.001$, **** $P < 0.0001$, # no detection). For b), g), h) and i), Student *t*-test was used to determine significance (* $P < 0.05$). For b), c), g), h) and i), Center line, median; box limits, upper and lower quartiles; plot limits, maximum and minimum in the boxplot. Source data are provided as a Source Data file.

Supplementary Tables:

LNA ASO ID	Type	Target region of SARS-CoV-2	Targeting Site	LNA ASO sequence with modification
5'-ASO#01	gapmer	5' leader sequence	GUUCUCUAAACGAAC	+G*+T*+T*/IMe-dC/C*G*T*T*T*A*G*A*G*+A*+A*+C
5'-ASO#02	gapmer	5' leader sequence	AUCUGUUCUCUAAACG	+C*+G*+T*T*T*A*G*A*G*A*A*C*+A*+G*+A*+T
5'-ASO#03	gapmer	5' leader sequence	GAUCUGUUCUCUAAAC	+G*+T*+T*T*A*G*A*G*A*A*C*A*G*+A*+T*+C
5'-ASO#04	gapmer	5' leader sequence	CUUGUAGAUCUGUUCUC	+G*+A*+G*A*A*C*A*G*A*T*C*T*A*C*+A*+A*+G
5'-ASO#05	gapmer	5' leader sequence	CUUGUAGAUCUGUUC	+G*+A*+A*C*A*G*A*T*C*T*A*C*+A*+A*+G
5'-ASO#06	gapmer	5' leader sequence	GAUCUCUUGUAGAUCUG	+C*+A*+G*A*T*C*T*A*C*A*A*G*A*G*+A*+T*+C
5'-ASO#07	gapmer	5' leader sequence	CGAUCUCUUGUAGAUC	+G*+A*+T*C*T*A*C*A*A*G*A*G*A*+T*+C*+G
5'-ASO#08	gapmer	5' leader sequence	AACUUUCGAUCUCUUG	+C*+A*+A*G*A*G*A*T*C*G*A*A*A*+G*+T*+T
5'-ASO#09	gapmer	5' leader sequence	GAUCUCUUGUAGAUC	+G*+A*+T*C*T*A*C*A*A*G*A*G*+A*+T*+C
5'-ASO#10	gapmer	5' leader sequence	ACCAACUUUCGAUCUC	+G*+A*+G*A*T*C*G*A*A*A*G*T*T*+G*+G*+T
5'-ASO#11	gapmer	5' leader sequence	AAACCAACCAACUUUC	+G*+A*+A*A*G*T*T*G*G*T*T*G*+G*+T*+T*+T
5'-ASO#12	gapmer	5' leader sequence	CAGGUAACAAACCAAC	+G*+T*+T*G*G*T*T*T*G*T*T*A*C*+C*+T*+G
5'-ASO#13	gapmer	5' leader sequence	AGGUAACAAACCAACC	+G*+G*+T*T*G*G*T*T*T*G*T*T*A*+C*+C*+T
5'-ASO#14	gapmer	5' leader sequence	UUCCCAGGUAACAAAC	+G*+T*+T*T*G*T*T*A*C*C*T*G*G*+G*+A*+A
5'-ASO#15	gapmer	5' leader sequence	ACCUUCCCAGGUAAC	+G*+T*+T*A*C*C*T*G*G*G*A*A*+G*+G*+T
5'-ASO#16	mixmer	5' leader sequence	GUUCUCUAAACGAAC	+G*+T*T*+C*G*+T*T*+T*A*+G*A*G*+A*+A*+C
5'-ASO#17	mixmer	5' leader sequence	AUCUGUUCUCUAAACG	+C*+G*+T*T*+T*A*+G*A*+G*+A*A*C*+A*+G*+A*+T
5'-ASO#18	mixmer	5' leader sequence	GAUCUGUUCUCUAAAC	+G*+T*T*+T*+A*G*+A*G*+A*A*+C*A*G*+A*+T*+C
5'-ASO#19	mixmer	5' leader sequence	CUUGUAGAUCUGUUCUC	+G*+A*G*+A*A*+C*+A*G+*A*T*+C*T*+A*C*+A*+A*+G
5'-ASO#20	mixmer	5' leader sequence	CUUGUAGAUCUGUUC	+G*+A*A*+C*A*+G*A*+T*C*+T*+A*C*A*+A*+G
5'-ASO#21	mixmer	5' leader sequence	GAUCUCUUGUAGAUCUG	+C*+A*G*A*+T*C*+T*+A*C*A*+A*G*+A*G*A*+T*+C
5'-ASO#22	mixmer	5' leader sequence	CGAUCUCUUGUAGAUC	+G*+A*+T*C*T*+A*+C*A*+A*+G*A*+G*A*+T*+C*+G
5'-ASO#23	mixmer	5' leader sequence	AACUUUCGAUCUCUUG	+C*+A*A*+G*A*+G*A*+T*+C*G*A*+A*+A*G*+T*+T
5'-ASO#24	mixmer	5' leader sequence	CGAUCUCUUGUAGAUC	+G*+A*T*+C*T*+A*+C*A*+A*G*+A*G*+A*T*+C*+G
5'-ASO#25	mixmer	5' leader sequence	ACCAACUUUCGAUCUC	+G*+A*G*+A*+T*C*+G*A*+A*+A*G*+T*T*G*+G*+T
5'-ASO#26	mixmer	5' leader sequence	AAACCAACCAACUUUC	+G*+A*A*+A*+G*T*+T*G*+G*+T*T*+G*G*+T*+T*+T
5'-ASO#27	mixmer	5' leader sequence	CAGGUAACAAACCAAC	+G*+T*T*G*+G*+T*T*+T*G*+T*+T*A*C*+C*+T*+G
5'-ASO#28	mixmer	5' leader sequence	AGGUAACAAACCAACC	+G*+G*T*+T*+G*G*T*+T*T*+G*T*+T*A*+C*+C*+T
5'-ASO#29	mixmer	5' leader sequence	UUCCCAGGUAACAAAC	+G*+T*T*+T*G*+T*T*+A*+C*C*+T*G*G*G*+A*+A
5'-ASO#30	mixmer	5' leader sequence	ACCUUCCCAGGUAAC	+G*+T*T*+A*+C*C*+T*G*+G*G*+A*+A*G*+G*+T
S-ASO#01	gapmer	Spike coding region	AUCAACUUACUCCUACU	+A*+G*+T*A*G*G*A*G*T*A*A*G*T*T*+G*+A*+T
S-ASO#02	gapmer	Spike coding region	CCCUACUUAUUGUUAAU	+A*+T*+T*+A*A*C*A*A*T*A*A*G*T*+A*+G*+G*+G
S-ASO#03	gapmer	Spike coding region	CAGACUAAUUCUCCUCG	+C*+G*+A*G*G*A*G*A*A*T*T*A*G*T*C*+T*+G
S-ASO#04	gapmer	Spike coding region	GUCCUAUAUAAUUCCG	+C*+G*+G*+A*A*T*T*A*T*A*T*A*G*+G*+A*+C
S-ASO#05	gapmer	Spike coding region	UCUGCUUUACUAAUGUC	+G*+A*+C*+A*T*T*A*G*T*A*A*A*G*C*+A*+G*+A
S-ASO#06	gapmer	Spike coding region	GGUGUUCUUACUGAGUC	+G*+A*+C*T*C*A*G*T*A*A*G*A*A*C*A*+C*+C
S-ASO#07	gapmer	Spike coding region	AUGUCCUUCCCUCAGUC	+G*+A*+C*+T*G*A*G*G*G*A*A*G*G*A*C*+A*+T
S-ASO#08	gapmer	Spike coding region	GCUUACUCUAAUAACUC	+G*+A*+G*+T*T*A*T*T*A*G*A*G*T*A*A*+G*+C
S-ASO#09	gapmer	Spike coding region	AGUUUAUGAUCCUUUGC	+G*+C*+A*A*A*G*G*A*T*C*A*T*A*+A*+A*+C*+T
S-ASO#10	gapmer	Spike coding region	UGAAUAUGUCUCUCAGC	+G*+C*+T*G*A*G*A*G*A*C*A*T*A*T*+T*+C*+A
S-ASO#11	gapmer	Spike coding region	CAGUGUGUUAAUCUUAC	+G*+T*+A*+A*G*A*T*T*A*A*C*A*C*A*+C*+T*+G
S-ASO#12	gapmer	Spike coding region	AAUCAUUACUACAGAC	+G*+T*+C*+T*G*T*A*G*T*A*A*T*+G*+A*+T*+T
S-ASO#13	gapmer	Spike coding region	GAACAAAUACUUCUAAC	+G*+T*+T*+A*G*A*A*G*T*A*T*T*T*+G*+T*+T*+C
S-ASO#14	gapmer	Spike coding region	UGAGAUUCUUGACAUUA	+T*+A*+A*+T*G*T*C*A*A*G*A*A*T*+C*+T*+C*+A
S-ASO#15	gapmer	Spike coding region	UGCUAAUCUUGCUGCUA	+T*+A*G*C*A*G*C*A*A*G*A*T*T*A*G*+C*+A
S-ASO#16	gapmer	Spike coding region	UGGUGAUAUUGCUGCUA	+T*+A*G*C*A*G*C*A*A*T*A*T*C*A*C*+C*+A
S-ASO#17	gapmer	Spike coding region	CACUUGACCCUCUCUCA	+T*+G*A*G*A*G*A*G*G*G*T*C*A*A*+G*+T*+G
U-ASO#01	mixmer	5'UTR regions of ORF1a	GAUGGAGAGCCUUGUC	+G*+A*C*+A*A*+G*G*+C*+T*C*+T*+C*C*+A*+T*+C
U-ASO#02	mixmer	5'UTR regions of ORF1a	GGUGUGACCGAAAGG	+C*+C*T*+T*T*+C*G*+G*T*+C*+A*C*+A*+C*+C
U-ASO#03	mixmer	5'UTR regions of ORF1a	AUCUAGGUUUCGUCCG	+C*+G*G*+A*C*+G*+A*A*+A*C*+C*+T*A*+G*+A*+T
U-ASO#04	mixmer	5'UTR regions of ORF1a	CAGCCGAUCAUCAGCAC	+G*+T*G*+C*T*+G*A*+T*+G*A*+T*+C*G*+G*C*+T*+G
U-ASO#05	mixmer	5'UTR regions of ORF1a	GGUUUCGUCCGUGUUG	+C*+A*A*+C*+A*C*+G*G*+A*+C*G*+A*A*+A*+C*+C
U-ASO#06	mixmer	5'UTR regions of ORF1a	CUGCAGGCUGCUUAC	+G*+T*A*+A*+G*C*+A*+G*C*+C*T*+G*C*+A*+G
U-ASO#07	mixmer	5'UTR regions of ORF1a	GUAACUCGUCUAUCUU	+A*+A*+G*A*+T*A*+G*A*+C*G*+A*G*+T*T*+A*+C
U-ASO#08	mixmer	5'UTR regions of ORF1a	CGUUGACAGGACACGA	+T*+C*+G*T*+G*T*+C*C*+T*G*+T*C*+A*A*+C*+G
U-ASO#09	mixmer	5'UTR regions of ORF1a	UAAUAACUAAUUACUGU	+A*+C*A*+G*T*+A*+A*T*+T*A*+G*T*+T*A*+T*+T*+A
U-ASO#10	mixmer	5'UTR regions of ORF1a	CUCACGCAGUAUAAU	+A*+T*+T*A*+T*A*+C*+T*G*+C*G*T*+G*+A*+G
U-ASO#11	mixmer	5'UTR regions of ORF1a	CUGCAUGCUUAGUGCA	+T*+G*C*+A*C*+T*+A*A*+G*+C*A*+T*G*+C*+A*+G
U-ASO#12	mixmer	5'UTR regions of ORF1a	GUGUGGCUGUCACUCGG	+C*+C*G*+A*+G*T*+G*A*+C*A*+G*C*+C*A*+C*+A*+C
U-ASO#13	mixmer	5'UTR regions of ORF1a	ACGAACUUUAAAAUCU	+A*+G*A*+T*T*+T*T*+A*+A*A*+G*T*+T*C*+G*+T
U-ASO#14	mixmer	5'UTR regions of ORF1a	GUAGAUCUGUUCUCUAA	+T*+T*A*+G*A*+G*+A*A*+C*A*+G*+A*T*+C*T*+A*+C
U-ASO#15	mixmer	5'UTR regions of ORF1a	CAACUUUCGAUCUCUU	+A*+A*+G*A*+G*A*+T*C*+G*+A*A*+A*+G*T*+T*+G
U-ASO#16	mixmer	5'UTR regions of ORF1a	CAGGUAACAAACCAAC	+G*+T*T*+G*G*+T*T*+T*+G*T*+T*T*+C*C*+T*+G
U-ASO#17	mixmer	5'UTR regions of ORF1a	AAGGUUUAUACCUUCC	+G*+G*A*+A*G*+G*T*+A*+T*A*+A*A*+C*C*+T*+T
F-ASO#01	mixmer	FSE region of ORF1a/b	CAAGAAAAGGACGAAG	+C*+T*T*+C*G*+T*+C*C*+T*T*+T*T*C*+T*+T*+G
F-ASO#02	mixmer	FSE region of ORF1a/b	UAAUUGUUGUCGCUUC	+G*+A*A*+G*+C*G*+A*+C*A*+A*+C*A*A*+T*+T*+A
F-ASO#03	mixmer	FSE region of ORF1a/b	CUAAAUUCCUAAAAAC	+G*+T*T*+T*T*+T*A*+G*G*+A*+A*T*T*+T*+A*+G
F-ASO#04	mixmer	FSE region of ORF1a/b	AAAGUAGCUGGUUUUG	+C*+A*A*+A*A*+C*+C*A*+G*+C*T*+A*C*+T*+T*+T
F-ASO#05	mixmer	FSE region of ORF1a/b	UGACAUCUACAAUGAU	+A*+T*+C*A*+T*T*+G*T*+A*G*+A*+T*G*+T*+C*+A
F-ASO#06	mixmer	FSE region of ORF1a/b	UCGUAUACAGGGCUUU	+A*+A*A*+G*+C*C*+C*+T*G*+T*+A*T*A*+C*+G*+A
F-ASO#07	mixmer	FSE region of ORF1a/b	GGCACUAGUACUGAUG	+C*+A*T*+C*+A*G*+T*A*+C*+T*A*+G*T*+G*+C*+C
F-ASO#08	mixmer	FSE region of ORF1a/b	UACACCGUGCGGCACA	+T*+G*T*+G*+C*C*+G*+C*A*+C*G*+G*T*+G*+T*+A
F-ASO#09	mixmer	FSE region of ORF1a/b	UAAGUGCAGCCCGUCU	+A*+G*A*+C*+G*G*+G*+C*T*+G*C*+A*C*+T*+T*+A
F-ASO#10	mixmer	FSE region of ORF1a/b	AAACGGGUUUGCGGUG	+C*+A*+C*C*+G*C*+A*A*+A*+C*C*+C*G*+T*+T*+T
F-ASO#11	mixmer	FSE region of ORF1a/b	AUGCACAAUCGUUUUU	+A*+A*A*+A*A*+C*+G*A*+T*+T*G*+T*G*+C*+A*+T
F-ASO#12	mixmer	FSE region of ORF1a/b	AUGCUUCAGUCAGCUG	+C*+A*G*+C*T*+G*A*+C*+T*G*+A*+A*G*+C*+A*+T
F-ASO#13	mixmer	FSE region of ORF1a/b	UCAACUCCGCGAACCC	+G*+G*G*+T*+T*C*+G*C*+G*G*+A*+G*T*+T*+G*+A
F-ASO#14	mixmer	FSE region of ORF1a/b	AUGGCUGUAGUUGUGA	+T*+C*A*+C*+A*A*+C*+T*A*+C*+A*G*C*+C*+A*+T
F-ASO#15	mixmer	FSE region of ORF1a/b	GGUAUGUGGAAAGGUU	+A*+A*+C*C*+T*T*+T*+C*C*+A*+C*A*+T*A*+C*+C
F-ASO#16	mixmer	FSE region of ORF1a/b	AGUCUGUACCGUCUGC	+G*+C*A*+G*A*+C*G*+G*T*+A*+C*A*+G*+A*+C*+T
F-ASO#17	mixmer	FSE region of ORF1a/b	ACACUUAAAAACAC	+G*+T*+G*T*+T*T*+T*T*+A*A*G*+T*+G*+T
F-ASO#18	mixmer	FSE region of ORF1a/b	ACCCUGUGGGUUUU	+A*+A*+A*A*+C*C*+C*A*+C*A*G*+G*+G*+T
F-ASO#19	mixmer	FSE region of ORF1a/b	CGCUUCCAAGAAAAGG	+C*+C*T*+T*T*+T*+C*T*+T*G*+G*+A*A*+G*+C*+G
F-ASO#20	mixmer	FSE region of ORF1a/b	AAAAACUAAUUGUUGU	+A*+C*A*+A*+C*A*+A*T*+T*A*+G*+T*T*+T*+T*+T
F-ASO#21	mixmer	FSE region of ORF1a/b	GUUUUGCUAAAUUCCU	+A*+G*G*+A*A*+T*T*+T*+A*G*+C*+A*A*+A*+A*+C
F-ASO#22	mixmer	FSE region of ORF1a/b	AAUGAUAAAGUAGCUG	+C*+A*G*+C*+T*A*+C*T*+T*T*+A*T*C*+A*+T*+T
F-ASO#23	mixmer	FSE region of ORF1a/b	GGCUUUUGACAUCUAC	+G*+T*A*+G*A*+T*G*+T*C*+A*A*+A*A*+G*+C*+C
F-ASO#24	mixmer	FSE region of ORF1a/b	CUGAUGUCGUAUACAG	+C*+T*G*+T*A*+T*A*+C*G*+A*C*+A*T*+C*+A*+G
F-ASO#25	mixmer	FSE region of ORF1a/b	GGCACAGGCACUAGUA	+T*+A*C*+T*+A*G*+T*G*+C*C*+T*G*T*+G*+C*+C
F-ASO#26	mixmer	FSE region of ORF1a/b	CCGUCUUACACCGUGC	+G*+C*A*+C*G*+G*T*+G*T*+A*+A*G*A*+C*+G*+G
F-ASO#27	mixmer	FSE region of ORF1a/b	GCGGUGUAAGUGCAGC	+G*+C*T*+G*C*+A*C*+T*T*+A*C*+A*C*+C*+G*+C
F-ASO#28	mixmer	FSE region of ORF1a/b	GUUUUUAAACGGGUUU	+A*+A*A*+C*C*+C*+G*T*+T*T*+A*+A*A*+A*+A*+C
F-ASO#29	mixmer	FSE region of ORF1a/b	CAGCUGAUGCACAAUC	+G*+A*T*+T*G*+T*G*+C*A*+T*+C*A*G*+C*+T*+G
F-ASO#30	mixmer	FSE region of ORF1a/b	GAACCCAUGCUUCAGU	+A*+C*T*+G*A*+A*+G*C*+A*T*+G*G*G*+T*+T*+C
F-ASO#31	mixmer	FSE region of ORF1a/b	UUGUGAUCAACUCCGC	+G*+C*G*+G*A*+G*T*+T*+G*A*+T*C*A*+C*+A*+A
F-ASO#32	mixmer	FSE region of ORF1a/b	AAGGUUAUGGCUGUAG	+C*+T*A*+C*A*+G*C*+C*A*+T*A*+A*C*+C*+T*+T
F-ASO#33	mixmer	FSE region of ORF1a/b	GUCUGCGGUAUGUGGA	+T*+C*C*+A*C*+A*T*+A*C*+C*G*+C*A*+G*+A*+C
F-ASO#34	mixmer	FSE region of ORF1a/b	AAACACAGUCUGUACC	+G*+G*T*+A*C*+A*+G*A*+C*T*+G*T*G*+T*+T*+T
F-ASO#35	mixmer	FSE region of ORF1a/b	GUGGGUUUUACACUUAA	+T*+T*A*+A*G*+T*+G*T*+A*A*+A*A*+C*C*+C*+A*+C
F-ASO#36	mixmer	FSE region of ORF1a/b	AAACGGGUUUGCGGUG	+C*+A*C*+C*G*+C*A*+A*+A*C*+C*C*G*+T*+T*+T
F-ASO#37	mixmer	FSE region of ORF1a/b	AUGCACAAUCGUUUUU	+A*+A*A*+A*A*+C*+G*A*+T*T*+G*T*G*+C*+A*+T
F-ASO#38	mixmer	FSE region of ORF1a/b	UGCUUCAGUCAGCUG	+C*+A*G*+C*T*+G*A*+C*T*+G*+A*A*+G*+C*+A
F-ASO#39	mixmer	FSE region of ORF1a/b	CUCCGCGAACCCA	+T*+G*G*+G*T*+T*+C*G*+C*G*+G*+A*+G
F-ASO#40	mixmer	FSE region of ORF1a/b	UUUAAACGGGUUUGCG	+C*+G*C*+A*A*+A*C*+C*+C*G*+T*T*T*+A*+A*+A
F-ASO#41	mixmer	FSE region of ORF1a/b	CUGAUGCACAAUCGUU	+A*+A*C*+G*A*+T*T*+G*T*+G*C*+A*T*+C*+A*+G
F-ASO#42	mixmer	FSE region of ORF1a/b	CCCAUGCUUCAGUCAG	+C*+T*G*+A*+C*T*+G*A*+A*G*+C*A*T*+G*+G*+G

"+" indicates locked nuceotides, "*" indicates phosphorothioate (PS) bond

Supplementary Table 1. Targeting sites and LNA ASOs used in *in vitro* screening.
To identify LNA ASOs exhibiting anti-viral efficacy, LNA ASOs targeting 5' leader sequences, 5' UTR region of ORF1a, FSE of ORF1a/b and Spike coding region were tested in cell-based screening assays in Huh-7 cells. *In vitro* Mixmer Control and *in vitro* Gapmer Control used for cell-based screening and *in vivo* Control LNA ASO used for animal tests are listed in Table S3.

Up-regulated Gene by 5'-ASO#26	Down-regulated Gene by 5'-ASO#26	Up-regulated Gene by in vivo Mixmer LNA ASO	Down-regulated Gene by in vivo Mixmer LNA ASO
Trim30c	Ear2	Muc5ac	Omd
Lrg1	Penk	Agr2	Gpr82
Sprr1a	Cpne5	Fcgbp	
Slc39a2	Gm12576	Cenpm	
2010005H15Rik	Ltc4s	Iqgap3	
Gadd45g	Cxcr1	Serpina3n	
Tmem171		Gadd45g	
Ccl17		Mcm5	
Itih4		Cdc6	
Chaf1a		Gp49a	
Serpina3n		Spon2	
Lcn2		Chek1	
Slc26a4		Ttk	
Adamts4		Tcf19	
Saa1		Chaf1a	
Trim30d		Cxcr1	
Xaf1		Pigr	
Rnf213		Rmi2	
Rtp4		Timp1	
Oas1b		Uhrf1	
Il18bp		Adamts4	
Trim30a		Lrg1	
Irgm1		Dscc1	
Herc6		Retnla	
Igtp		Tnfrsf9	
Slfn8		Lcn2	
Iigp1		Slc26a4	
Gbp6		Cxcl1	
Phf11b		Clca3	
Ifi27l2a		Chil4	
Gm4951		Ly6i	
Ccl4		Saa3	
Cd274		Saa1	
Nlrc5		Cxcl5	
Irgm2			
Phf11c			
Ccl22			
Tgtp2			
Gbp3			
Hap1			
Tap1			
Ifi47			
Areg			
Stat1			
Apol7a			
Stat2			
Socs3			
Adar			
Parp9			
Dtx3l			
Gm6548			
Eif2ak2			
Lgals3bp			
Parp14			
Ifih1			
Ddx58			
Ddx60			
Ppa1			
Lgals9			
Apod			
Misp			
Olfr56			
Ccl12			
Rgs1			
Ifi205			
BC023105			
Phf11a			
Mnda			
Rsad2			
Il6			
Phf11d			
Cmpk2			
Gm14446			
Usp18			
Dhx58			
Oas2			
Ifi204			
Oas1a			
Gm12250			
Apol9b			
I830012O16Rik			
Oasl2			
Ifi44			
Zbp1			
Saa3			
Slfn9			
Ccl2			
Ccl7			
Mx2			
Irf7			
Oas3			
Oas1g			
Tgtp1			
Apol9a			
Cxcl5			
Ifit3			
Gm5431			
Ifit2			
Cxcl9			
Oasl1			
Cxcl10			
Ifit1			
Isg15			
Mx1			
Ly6i			

Supplementary Table 2. Differential expressed genes in mouse lung after LNA ASO treatment. The gene list of significantly up- and down-regulated (> 2-fold, FDA < 0.01) genes in 5'-ASO#26-treated or Mixmer Control LNA ASO-treated mice when compared with Saline Control.

Oligo list

Name	Forward oligo	Reverse oligo	Assay
Nucleocapsid (F/R)	5’-GACCCCAAAATCAGCGAAAT-3’	5’-TCTGGTTACTGCCAGTTGAATCTG-3’	qPCR
Spike (F/R)	5’-GTCCTTCCCTCAGTCAGCAC-3’	5’-ATGGCAGGAGCAGTTGTGAA-3’	qPCR
human r18S (F/R)	5’- GTAACCCGTTGAACCCCATT-3’	5’- CCATCCAATCGGTAGTAGCG-3’	qPCR
mouse r18S (F/R)	5’- GCAATTATTCCCCATGAACG -3’	5’- GGCCTCACTAAACCATCCAA -3’	qPCR
gBlock (F/R)	5’-TAATACGACTCACTATAGGGATTAAAGGTTTATACCTTCCCAGGTAAC-3’	5’-TCGTTGAAACCAGGGACAAG-3’	gBlock amplification
5'-leader (F/R)	5’-GGGATTAAAGGTTTATACCTTCCC-3’	5’-TCGTTGAAACCAGGGACAAG-3’	5'-leader amplification
anti-5'-ASO#26	5'-+A+A+AC+CA+A+CC+AA+C+TT+T+C/3AlexF488N/-3'	NA	FISH
In vitro Mixmer Control	+T*+G*C*+A*G*+A*T*+A*T*+G*C*+G*G*+G*+T	NA	for in vitro assay
In vitro Gapmer Control	+T*+G*+C*A*G*A*T*A*T*G*C*G*+G*+G*+T	NA	for in vitro assay
In vivo Control LNA ASO	+C*G*+T*T*+A*G*+A*+T*T*+A*+C*/iMe-dC/*G*+C*+G	NA	for in vivo assay

"+" indicates locked nuceotides, "*" indicates phosphorothioate (PS) bond, "/iMe-dC/" indicates 5-Methyl deoxyCytidine

Supplementary Table 3. Oligonucleotide (Oligo) list for different assays.
A DNA or Probe list for RT-qPCR, regular DNA amplification and FISH assay.
