# Supplementary material for: An intranasal ASO therapeutic targeting SARS-CoV-2
Source: Nat Commun. 2022 Aug 3;13:4503. doi: 10.1038/s41467-022-32216-0 (PMC9349213; doi:10.1038/s41467-022-32216-0)
Supplement: Supplementary file 2 — Reporting Summary [file 41467_2022_32216_MOESM2_ESM.pdf]

nature portfolio

Corresponding author(s): Anders M. Näär

Last updated by author(s): Jul 1, 2022

Reporting Summary

Nature Portfolio wishes to improve the reproducibility of the work that we publish. This form provides structure for consistency and transparency in reporting. For further information on Nature Portfolio policies, see our Editorial Policies and the Editorial Policy Checklist.

Statistics

For all statistical analyses, confirm that the following items are present in the figure legend, table legend, main text, or Methods section.

n/a	Confirmed	
☐	☒	The exact sample size (n) for each experimental group/condition, given as a discrete number and unit of measurement
☐	☒	A statement on whether measurements were taken from distinct samples or whether the same sample was measured repeatedly
☐	☒	The statistical test(s) used AND whether they are one- or two-sided<br>*Only common tests should be described solely by name; describe more complex techniques in the Methods section.*
☐	☒	A description of all covariates tested
☐	☒	A description of any assumptions or corrections, such as tests of normality and adjustment for multiple comparisons
☐	☒	A full description of the statistical parameters including central tendency (e.g. means) or other basic estimates (e.g. regression coefficient) AND variation (e.g. standard deviation) or associated estimates of uncertainty (e.g. confidence intervals)
☐	☒	For null hypothesis testing, the test statistic (e.g. F, t, r) with confidence intervals, effect sizes, degrees of freedom and P value noted<br>*Give P values as exact values whenever suitable.*
☒	☐	For Bayesian analysis, information on the choice of priors and Markov chain Monte Carlo settings
☒	☐	For hierarchical and complex designs, identification of the appropriate level for tests and full reporting of outcomes
☒	☐	Estimates of effect sizes (e.g. Cohen's d, Pearson's r), indicating how they were calculated

Our web collection on statistics for biologists contains articles on many of the points above.

Software and code

Policy information about availability of computer code

Data collection	BD FACSDiva 8.0.1 was used for cell analyzer data collection, G*Power 3.1 was used for size evaluation
Data analysis	Prism 8 was used in this paper for t-test and one-way or two-way ANOVA test. For RNA-seq analysis, STAR aligner v2.5.4a [PMID 23104886] was used to map sequencing reads to transcripts in the mouse mm10 reference genome. Read counts for individual transcripts were produced with HTSeq-count v0.12.4 [PMID 25260700], followed by the estimation of expression values and detection of differentially expressed transcripts using EdgeR v3.26.8 [PMID 19910308]. Gene set enrichment analysis was performed by GSEA 3.0 [PMID 12808457] against MSigDB v7.1. BD FACSDiva 8.0.1 and FlowJo v10.8.1 were used for cell analyzer data analysis.

For manuscripts utilizing custom algorithms or software that are central to the research but not yet described in published literature, software must be made available to editors and reviewers. We strongly encourage code deposition in a community repository (e.g. GitHub). See the Nature Portfolio guidelines for submitting code & software for further information.

Data

Policy information about availability of data

All manuscripts must include a data availability statement. This statement should provide the following information, where applicable:

- Accession codes, unique identifiers, or web links for publicly available datasets
- A description of any restrictions on data availability
- For clinical datasets or third party data, please ensure that the statement adheres to our policy

All data are available in the manuscript and associated files. Source data is provided with this paper. The RNA-seq data in this study has been uploaded to GEO (GSE174382) and publicly released. The link of other published dataset (GSE154104) is also listed in the paper.

Field-specific reporting

Please select the one below that is the best fit for your research. If you are not sure, read the appropriate sections before making your selection.

[x] Life sciences [ ] Behavioural & social sciences [ ] Ecological, evolutionary & environmental sciences

For a reference copy of the document with all sections, see nature.com/documents/nr-reporting-summary-flat.pdf

Life sciences study design

All studies must disclose on these points even when the disclosure is negative.

Sample size	In animal trial, to evaluate the biologically meaningful viral repressive effect, we expected to see at least a 75% repressive effect of LNA ASO group when compared with control. So we estimated that the mean in control group is 4-fold larger than that in LNA ASO group and we also estimated the SD of each group is about 20% of mean of each group. The sample size calculated by G*Power 3.1 [using T-tests, Mean: Difference between two independent means (two groups) option in the G*Power 3.1 software] indicated that to achieve the significance (p <0.05), 3 samples in each group is required. Therefore, we used 5 mice in each group to have extra sensitivity.
Data exclusions	No data was excluded
Replication	All in vitro experiments were repeated at least twice and for in vivo experiment and showed good consistency, all in vivo experiments conducted in C57BL/6J, K18-hACE2 mice and hamsters with SARS-CoV-2 WA1 and D.1.617.2 strain were repeated twice with good consistency and the other variants were only tested once in K18-hACE2 mice or hamsters. For Remdesivir tests in C57BL/6J Ces1c-/- mice were only tested once due to the limited number of animals.
Randomization	For mouse experiment, K18-ACE2 mice were bred in-house, and mice of same age were randomly assigned to control or treatment group, as long as the male/female ratio was same between the two conditions. Ces1-/- mice and C57BL/6J wild-type mice of same age were purchased from Jackson Laboratory. For hamsters experiment, male golden Syrian hamsters of same age were purchased from Charles River Labs, and then hamsters were randomly assigned to control or treatment groups. For cell-based assays, the treatments or procedures were not randomized since all experiments were carried in 24-well plates or plates with larger well size and there was no significant margin effect as observed with 96-well plates.
Blinding	Investigators were not blinded since many experiments need to be carried out in the BSL-3 lab with very limited personnel capacity, which means that one investigator has to conduct the whole experiment alone in BSL-3 and for safety concerns, we prefer to let investigators inside BSL-3 know as many details as possible so they can have their best judgment to evaluate urgent circumstance (leakage, spilling etc.). Meanwhile, sample collection in BSL-3 and sample process out of BSL-3 were conducted by different individuals to partially achieve blinding.

Reporting for specific materials, systems and methods

We require information from authors about some types of materials, experimental systems and methods used in many studies. Here, indicate whether each material, system or method listed is relevant to your study. If you are not sure if a list item applies to your research, read the appropriate section before selecting a response.

Materials & experimental systems

n/a	Involved in the study	
[ ]	[x]	Antibodies
[ ]	[x]	Eukaryotic cell lines
[x]	[ ]	Palaeontology and archaeology
[ ]	[x]	Animals and other organisms
[x]	[ ]	Human research participants
[x]	[ ]	Clinical data
[x]	[ ]	Dual use research of concern

Methods

n/a	Involved in the study	
[x]	[ ]	ChIP-seq
[ ]	[x]	Flow cytometry
[x]	[ ]	MRI-based neuroimaging

Antibodies

Antibodies used	Rabbit monoclonal CD3 primary antibody[SP7] (Abcam, ab16669, lot: not available), B220 primary antibody[RA3-6B2] (Novus, NB100-77420, lot: not available), rabbit monoclonal SARS-CoV-2 (COVID-19) nucleocapsid primary antibody [HL448] (GeneTex, GTX635686, not available), rat anti-mouse Gr1/Ly-6G-Alexa Fluor 594[1A8] (Novus, NBP2-53131AF594, lot: A-1-072921-AF594), rat anti-mouse CD11b-FITC[M1/70] (R&D System, FAB1124F, lot:LBQ0309121) and rat anti-mouse-F4/80-APC[# 521204] (R&D System,FAB5580A, lot:ABGS0221081), mouse IgG(Abcam, ab37355, lot:GR3233337-2)
Validation	No validation statement from Abcam for CD3 primary antibody. B220 primary antibody was validated by orthogonal strategy based on the information stated on Novus website. SARS-CoV-2 nucleocapsid primary antibody was validated by lateral flow assay based on the information stated on GeneTex website. Furthermore, IHC staining validation of CD3 and B220 were conducted in mouse spleen tissue and IHC staining validation of SARS-CoV-2 nucleocapsid antibody was conducted in lung of SARS-CoV-2-infected mouse. The

IHC staining validation was carried out by HistoWiz Inc. Rat anti-mouse Gr1/Ly-6G-Alexa Fluor 594 antibody was not validated by the vendor, but the FITC version of this antibody was validated in the single cell suspension isolated from spleen of an IL1-R KO mouse by other customers on Novus website. Meanwhile, in this study, we used LPS intranasal administrated mice as a positive control for detecting neutrophils in lung since it is known that intranasal administration of LPS will enhance the amount of neutrophils in lung (PMID: 11076798,22355412 etc.) and we indeed detected a significantly increased cell population in LPS-treated mice by using Rat anti-mouse Gr1/Ly-6G-Alexa Fluor 594 antibody. Therefore, Rat anti-mouse Gr1/Ly-6G-Alexa Fluor 594 antibody should be considered as a validated antibody. Rat anti-mouse CD11b-FITC was validated in mouse peripheral blood cells by flow cytometry on R&D System website and rat anti-mouse-F4/80-APC antibody was validated in raw264.7 mouse cell line by flow cytometry on R&D System website. mouse IgG was validated by ELISA showed on Abcam website.

Eukaryotic cell lines

Policy information about cell lines

Cell line source(s)	Vero E6 from ATCC, Vero 81 received from Mary Kate Morris of California Department of Public Health (CDPH) as a kind gift (orignally from ATCC), Vero E6 TMPRSS2/hACE was received from Biodefense and Emerging Infections Research Resources Repository (BEI), Huh-7 from Dr. Eva Harris who received them as a kind gift from Dr. Asim Dasgupta.
Authentication	No authentication procedure
Mycoplasma contamination	Cell lines were not tested for mycoplasma contamination
Commonly misidentified lines (See ICLAC register)	no commonly misidentified cell lines were used in the study

Animals and other organisms

Policy information about studies involving animals; ARRIVE guidelines recommended for reporting animal research

Laboratory animals	8 ~10 weeks-old male/female K18-hACE2-mice e(B6.Cg-Tg(K18-ACE2)2Prlmn/J), and 8 weeks-old 8 ~10 weeks-old male/female C57BL/6J Ces1c-/- [B6.Cg-Ces1ctm1.1Loc/J], 8 weeks-old male C57BL/6J, and 8-weeks old male LVG golden Syrian hamsters
Wild animals	no wild animals were used in the study
Field-collected samples	no field collected samples were used in the study
Ethics oversight	All procedures involving the use of mice and hamsters were approved by the University of California, Berkeley Institutional Animal Care and Use Committee. All protocols conform to federal regulations, the National Research Council Guide for the Care and Use of Laboratory Animals, and the Public Health Service Policy on Humane Care and Use of Laboratory Animals.

Note that full information on the approval of the study protocol must also be provided in the manuscript.

Flow Cytometry

Plots

Confirm that:

- [x] The axis labels state the marker and fluorochrome used (e.g. CD4-FITC).
- [x] The axis scales are clearly visible. Include numbers along axes only for bottom left plot of group (a 'group' is an analysis of identical markers).
- [x] All plots are contour plots with outliers or pseudocolor plots.
- [x] A numerical value for number of cells or percentage (with statistics) is provided.

Methodology

Sample preparation	Mice treated intranasally with lipopolysaccharides (60 µg in 40 µl saline, LPS from Escherichia coli O111:B4, Sigma) for one day were used as positive control. One day after the last administration, mice were sacrificed under anesthesia with 3% isoflurane, then BALF samples were collected by making an incision in the trachea and washing the lungs 5 times with 1 mL PBS, and repeating again with additional 1 ml PBS. Cells collected from BALF were incubated in 100ul 1xRBC lysis buffer (BioLegend) on ice for 10 min, and then quenched by adding 1 ml PBS. Cells from each sample were resuspended in 100 µl Stain Buffer (554656, BD Pharmingen) and incubated for 15 min on ice with 1 µg mouse IgG (ab37355, Abcam) for Fc blocking. Then cells were incubated with primary monoclonal antibodies including rat anti-mouse Gr1/Ly-6G-Alexa Fluor 594 (Novus, 1:200), rat anti-mouse CD11b-FITC (R&D System,1:200) and rat anti-F4/80-APC (R&D System,1:200). Stained cells were resuspended in 500 µl stain Buffer (554656, BD Pharmingen) for cell analyzer.
Instrument	FACS LSR Fortessa X20 (BD Biosciences)
Software	Flowjo and BD FACSDiva
Cell population abundance	Cells samples under the "low" flow mode of FACS LSR Fortessa X20 (BD Biosciences) showed about ~100 events/s

Gating strategy	All events were pre-gated by FSC and SSC to make sure >95% populations were showed in the scales and cell doublets were excluded by FSC-A/H and validated by SSC-A/H. The Saline control group was considered as negative control for Gr1/Ly-6G (Filter:Texas Red), CD11b (Filter:FITC) and F4/80 (Filter: APC) and the main population distribution (>99% of pre-gated population) of Saline control group was considered as "negative" area and any cells above the boundary would be considered as "positive".

☒ Tick this box to confirm that a figure exemplifying the gating strategy is provided in the Supplementary Information.
